# The 1997 Mars Pathfinder Spacecraft Landing Site: Spillover Deposits from an Early Mars Inland Sea

**DOI:** 10.1038/s41598-019-39632-1

**Published:** 2019-02-25

**Authors:** J. A. P. Rodriguez, V. R. Baker, T. Liu, M. Zarroca, B. Travis, T. Hui, G. Komatsu, D. C. Berman, R. Linares, M. V. Sykes, M. E. Banks, J. S. Kargel

**Affiliations:** 1 Planetary Science Institute, 1700 East Fort Lowell Road, Suite 106, Tucson, AZ 85719-2395 USA; 2 Department of Hydrology & Atmospheric Sciences, University of Arizona, Tucson, AZ 85721 USA; 3 External Geodynamics and Hydrogeology Group, Department of Geology, Autonomous University of Barcelona, 08193 Bellaterra, Barcelona, Spain; 4 International Research School of Planetary Sciences, Università D’Annunzio, Viale Pindaro 42, 65127 Pescara, Italy; 5 NASA Goddard Space Flight Center, Goddard, MD 20771 USA

**Keywords:** Geomorphology, Hydrology

## Abstract

The Martian outflow channels comprise some of the largest known channels in the Solar System. Remote-sensing investigations indicate that cataclysmic floods likely excavated the channels ~3.4 Ga. Previous studies show that, in the southern circum-Chryse region, their flooding pathways include hundreds of kilometers of channel floors with upward gradients. However, the impact of the reversed channel-floor topography on the cataclysmic floods remains uncertain. Here, we show that these channel floors occur within a vast basin, which separates the downstream reaches of numerous outflow channels from the northern plains. Consequently, floods propagating through these channels must have ponded, producing an inland sea, before reaching the northern plains as enormous spillover discharges. The resulting paleohydrological reconstruction reinterprets the 1997 Pathfinder landing site as part of a marine spillway, which connected the inland sea to a hypothesized northern plains ocean. Our flood simulation shows that the presence of the sea would have permitted the propagation of low-depth floods beyond the areas of reversed channel-floor topography. These results explain the formation at the landing site of possible fluvial features indicative of flow depths at least an order of magnitude lower than those apparent from the analyses of orbital remote-sensing observations.

## Introduction

**Figure 1:**
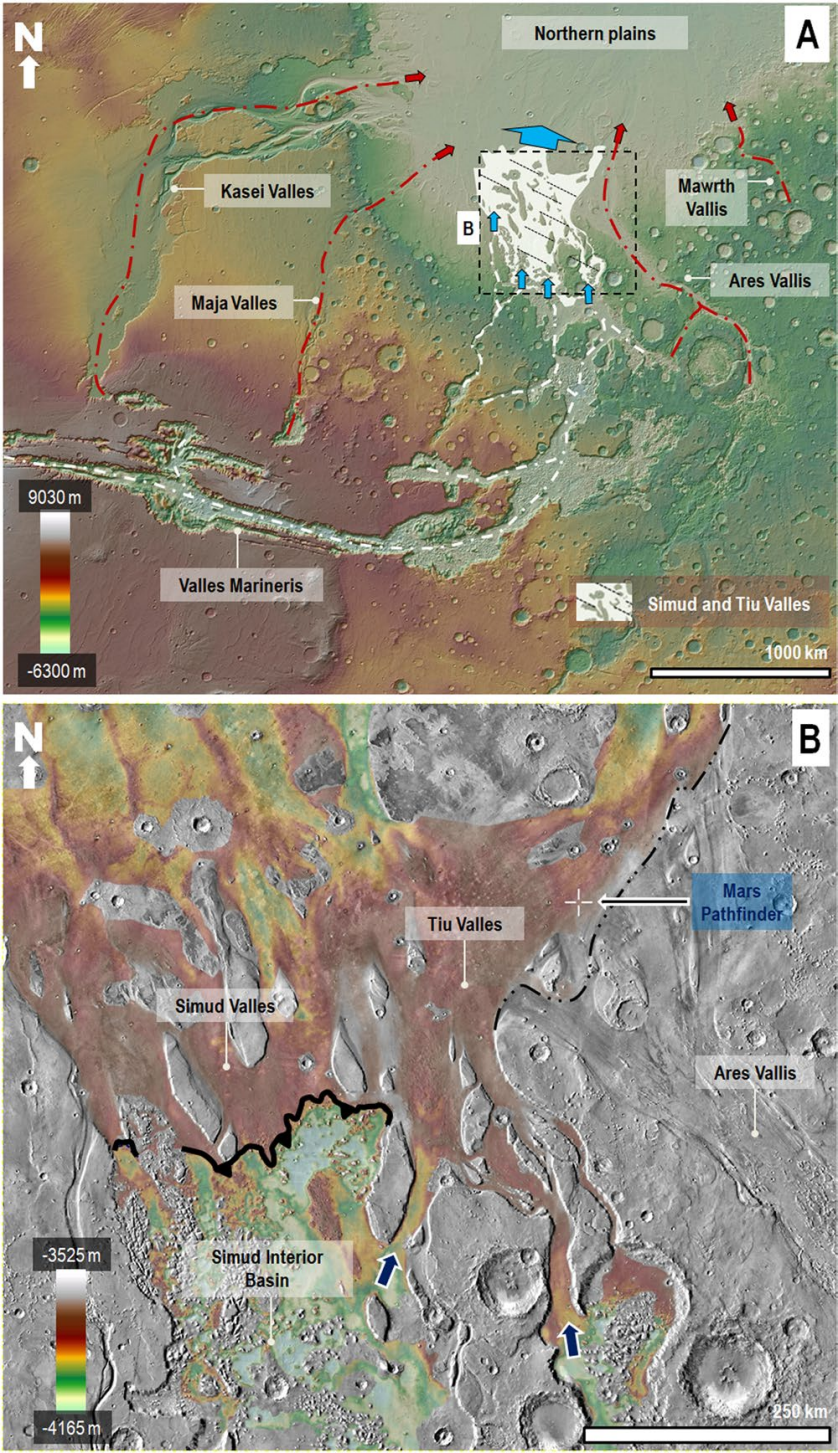
**(A)** View of Mars’ western hemisphere centered at 2°20′34″N, 49°51′57″W. The dashed white lines trace the planet’s largest canyon network, which includes the circum-Chryse outflow channels and Valles Marineris. This network converges into the northern lowlands (blue arrows) via Simud and Tiu Valles (area highlighted in white). The red lines and arrows indicate other circum-Chryse outflow channels, which are not part of the network. The base image for panel A is a color-coded shaded-relief Mars Orbiter Laser Altimeter (MOLA) digital elevation model (DEM) (460 m/pixel). Credit: MOLA Science Team, MSS, JPL, NASA. **(B)** View showing the topographic transition between the floors of the Simud Interior Basin (SIB) and the Simud/Tiu Valles system. See panel A for the view’s context and location. We removed from the DEM prominent topographic features such as impact craters, chaotic terrains, and teardrop-shaped islands to better resolve the broad-scale elevation changes that characterize the transition. The contact between the SIB and Simud Valles forms a south-facing break in slope that closely follows an elevation of −3,800 m (indicated by an indented black line). On the other hand, the contact between the SIB and Tiu Valles consists of two canyons (blue arrows) that exhibit reversed topographic gradients with  approximate overflow elevations at −3,700 m (western canyon) and −3,550 m (eastern canyon). The contact between Ares and Tiu Valles is an erosional unconformity (dashed black line)^3,14^. The crosshair symbol shows the location of the Mars Pathfinder landing site. The elevation ranges are from MOLA-extracted topography (460 m/pixel). Credit: MOLA Science Team, MSS, JPL, NASA. The base image for panel B is part of a Thermal Emission Imaging System (THEMIS) Day IR (infrared) 100 m Global Mosaic (http://www.mars.asu.edu/data/). Credit: Christensen *et al*.^61^. We produced the mosaic areas and maps in this figure using Esri’s ArcGIS^®^ 10.3 software (http://www.esri.com/software/arcgis).

On July 4, 1997, the Mars Pathfinder touched down on the lower reaches of Tiu and Ares Valles^1,2^ (Fig. 1), two of Mars’ largest outflow channels^3^. Numerous remote-sensing investigations of these outflow channels have linked their origin primarily to large-scale erosion by episodically occurring^4–7^ cataclysmic floods^8,9^. The floods were estimated to have had typical depths ranging from ~30 to ~300 m and flow velocities of up to ~30 m/s^10^. However, during channel-filling discharges, these values may have peaked at ~985 m and ~150 m/s, respectively^11–13^. Erosional vestiges of these cataclysmic floods, detected by orbital imaging, include sizeable teardrop-shaped islands^3,11,14,15^ and streamlined hills^1,2^. The regional context for this cataclysmic flooding shows that it originated from areas located several hundred^4–7^ to several thousand^4,5,13^ kilometers upstream from the Pathfinder site.

Detailed geological mapping shows that the youngest cataclysmic flood flows ensued from Tiu Valles, that they truncated the lowest reaches of Ares Vallis, and that their most recent manifestations resurfaced the Pathfinder site^3,14^ (Fig. 1B; see supplementary methodology for mapping approach). The Pathfinder’s camera imaged probable flood deposits in the vicinity of the lander, including rounded/semi-rounded pebbles, cobbles, and boulders, some of which show potential imbrication^1^. Using the size of the largest boulders and the inferred regional slope values, in combination with empirical relationships derived from terrestrial floods^16–18^, Smith *et al*.^2^ computed flow velocities of ~8 m/s, and flow depths ranging from ~10 to ~20 m. These values are an order of magnitude or so lower than inferred for the regional cataclysmic flooding peaks noted above. Moreover, the Smith *et al*.^2^ characterization of the size distribution and angularity of the sediments shows that nearby outcrops comprise their likely source areas.

These results starkly contrast with the much more massive flooding with distal sources that has been reconstructed from the orbital remote-sensing characterizations of these and other outflow channels^10–12^. However, the latter estimates mostly agree with values characteristic of ancient megaflooding in Iceland and Washington State, respectively produced by sub-glacier discharges and ice-dammed glacial lake failures^9,19,20^.

The presence of hundreds of kilometers of reversed gradient channel-floor areas leading to lower Simud and Tiu Valles^21–25^ indicates that the previously proposed waning stages in a cataclysmic flood^11^ cannot explain this discrepancy (Fig. 1B). This topographic configuration would have necessarily restricted floods capable of reaching the Pathfinder site to those with stages that gained sufficient elevations to exceed the total relief present in the reversed gradient floors.

A south-facing break in slope at a planetary elevation of −3,800 m comprises the lowest elevation spillover pathway connecting these reversed channel areas to the lower reaches of Simud Valles^24,25^ (Fig. 1B). Floodwaters retained below this elevation are hypothesized to have generated paleolakes^21–25^. However, the full extent of the consequential inundations remains unknown.

**Figure 2:**
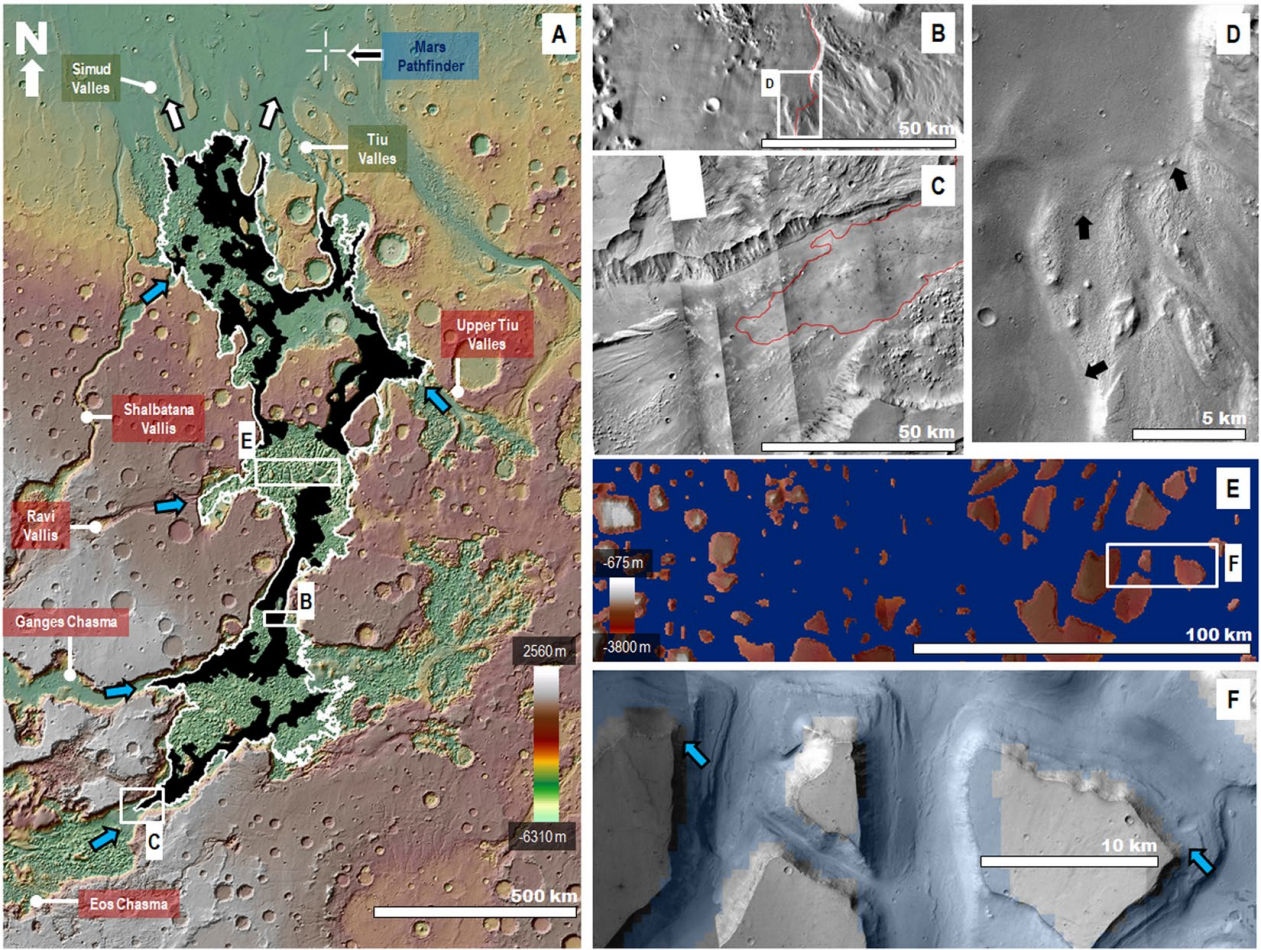
**(A)** View of the southern circum-Chryse region centered at 0°29′11″N, 34°0′22″W, including the SIB’s drainage divide at an elevation of −3,800 m (white line). The black areas indicate the distribution of smooth sedimentary mantle material, which has upper reaches bounded by  the divide topography. The blue arrows and boxes with a red background show the SIB’s inflow routes from both eastern VM and numerous outflow channels. The boxes with a green background identify Simud and Tiu Valles, which comprise the only outflow routes from the basin (white arrows). The crosshair symbol shows the location of the Mars Pathfinder landing site. The base image for panel A is a color-coded shaded-relief MOLA DEM (460 m/pixel). Credit: MOLA Science Team, MSS, JPL, NASA. **(B** and **C)** Close-up views of SIB contacts with adjoining, more elevated, outflow channel scoured floors. The red line identifies the basin’s divide at the −3,800 m elevation and closely traces the upper distribution of its smooth sedimentary unit. The base image for panel B is part of a THEMIS Day IR (infrared) 100 m Global Mosaic (http://www.mars.asu.edu/data/). Credit: Christensen *et al*.^61^. The image in panel C is part of a Context Camera (CTX) mosaic (~6 m/pixel). Credit: NASA/JPL. The license terms can be found at pds-imaging.jpl.nasa.gov/portal/mro_mission.html. **(D)** Close-up viewon panel B showing the sedimentary unit’s embayment of adjoining bedforms (black arrows). Panel D is a part of a CTX (~6 m/pixel) mosaic. Credit: NASA/JPL. The license terms can be found at pds-imaging.jpl.nasa.gov/portal/mro_mission.html. **(E)** View showing the distribution of mesas within central Hydraotes Chaos that stand above the −3,800 m elevation level (blue mask). The image is a combined elevation color-coded and shaded-relief MOLA DEM (460 m/pixel; credit: MOLA Science Team, MSS, JPL, NASA) over a CTX mosaic (~6 m/pixel; credit: NASA/JPL). The license terms can be found at pds-imaging.jpl.nasa.gov/portal/mro_mission.html. **(F)** Close-up view on panel E showing how the upper distribution of the terraces flanking the mesas closely follows the −3,800 m elevation level (surfaces highlighted in blue are at ≤−3,800 m elevation). The image is a part of a CTX mosaic (~6 m/pixel). Credit: NASA/JPL). The license terms can be found at pds-imaging.jpl.nasa.gov/portal/mro_mission.html. The CTX mosaic underlies a transparent MOLA-generated blue mask. Credit: MOLA Science Team, MSS, JPL, NASA. We produced the mosaic areas and maps in this figure using Esri’s ArcGIS^®^ 10.3 software (http://www.esri.com/software/arcgis).

Here, we show that this drainage divide encloses a vast basin located within the deepest areas of the outflow channel systems located south of Tiu and Simud Valles (Fig. 2A). This basin, which we refer to as the Simud Interior Basin (SIB, 15°41′N to 13°18′S), includes extensive plains as well as numerous interior mesas, some of which have tops reaching as much as several hundred meters above the −3,800 m elevation level. The SIB occupies a total surface area of ~430,000 km^2^ (approximately equivalent to that of California), of which 323,000 km^2^ lies below the divide. The basin encloses a total cavity volume of ~80,000 km^3^, which is similar to that of the Caspian Sea, Earth’s most extensive inland water body (see supplementary methodology for approach on volumetric characterizations).

Martian outflow channel activity appears to have been episodic and spanned from the Late Noachian^26–29^ to the Early Amazonian^7,14,30–35^. However, numerous mapping investigations indicate that outflow channel activity peaked during the Late Hesperian (~3.4 Ga)^3,4,14^, which is also the model age that our impact crater count statistics on the SIB’s surface indicate (Fig. S1; see supplementary methodology for approach on surface age determinations).

A texturally smooth sedimentary unit (Fig. 2A), which overlaps and embays the floors of numerous outflow channels connected to the basin’s periphery (Fig. 2B–D), extensively covers the SIB’s interior. This stratigraphic superposition indicates that the unit’s emplacement occurred following peak flooding activity during the Late Hesperian. An equipotential elevation corresponding to the −3,800 m elevation drainage divide delimits the uppermost reaches of these materials (Figs 2A; S2). The SIB’s entire inundation due to water ponding and subsequent complete evaporation can best account for the basin’s preservation as well as for the presence of sedimentary mantles with uppermost reaches that match the drainage divide’s elevation. Proposed alternatives invoke outflow channel histories dominated by debris flows^5,36,37^ and lava flows^38–40^. While highly fluidized debris flows and lavas could have also ponded up to the drainage divide, these fluid-flow processes would have buried the SIB (Fig. S3).

**Figure 3:**
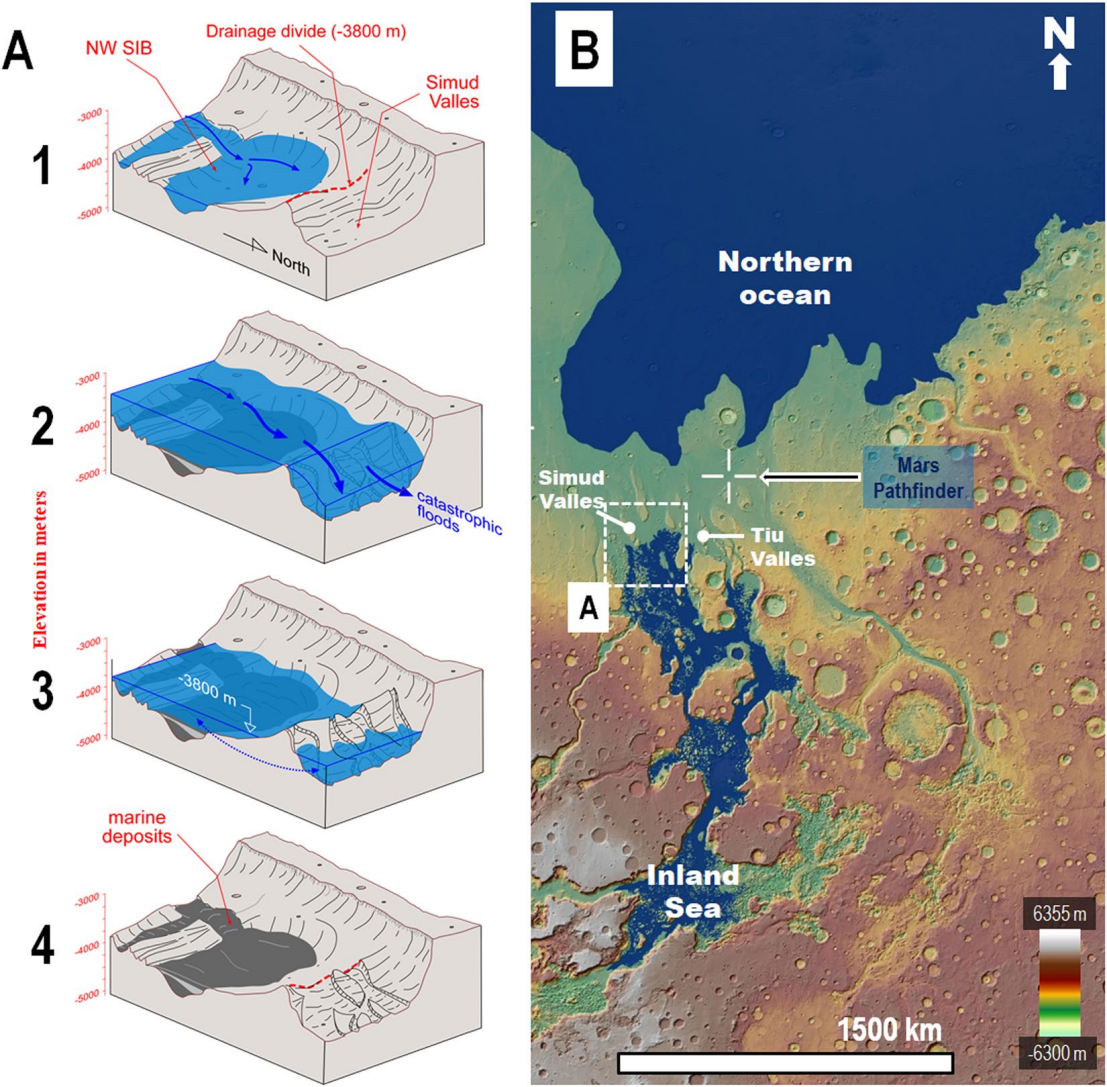
**(A)** Perspective sketches depicting the paleohydrological reconstruction of the SIB’s northwestern part and adjoining areas of Simud Valles. Panel (B) shows the sketched region’s context and location. Notice that the north direction is towards the bottom right. (1) View of an inland sea margin within the northwestern part of the SIB produced by the progressive impoundment of cataclysmic floods below the drainage divide at the −3,800 m elevation separating the basin and Simud Valles. (2) Floods propagating over the sea produce spillover marine discharges onto Simud and Tiu Valles, which reach the Pathfinder landing site and produce an ocean within the northern plains. (3) Following the cataclysmic floods, an inland sea and a northern plains ocean form. Both marine bodies share the same upper shoreline levels at the −3,800 m elevation, indicating the presence of an interconnected groundwater table at that time (double-headed arrow). (4) Extensive marine sedimentary deposits, with an upper distribution limited by the drainage divide, are left behind within the SIB following the sea’s disappearance. (**B**) Paleogeographic reconstruction including the two marine bodies formed after the cataclysmic floods (#3 in Panel A). The Pathfinder landing site (crosshair symbol) is located on an enormous spillway that connected the inland sea and the northern ocean. The blue mask over the digital elevation model corresponds to the reconstructed extents of the inland sea and the northern plains ocean. The base map is a MOLA digital elevation model (460 m/pixel) centered at 5°31′17″N, 30°51′24″W. Credit: MOLA Science Team, MSS, JPL, NASA.

Consequently, regional outflow channel activity must have resulted in the formation of an inland sea within the SIB’s confines (Fig. 3A, sketches 1–3; Fig. 3B). In this context, we interpret the texturally smooth sedimentary unit to be mostly composed of marine sediments overlying catastrophic flood deposits emplaced during the early inundation phases. Lithologically, the sediments are expected to be diverse because the floods would have transported materials eroded from the floor, wall and subsurface stratigraphy of multiple locations in southern circum-Chryse and Valles Marineris (VM), an immense canyon with an extension equivalent to the width of the continental United States. Our observations, however, do not exclude the possibility that older debris flows^5,36,37^ and lava flows^38–40^, discharged via the regional outflow channel system, exist within the stratigraphic record buried beneath these marine sediments.

The floors of VM open east into the broader zones of the southern circum-Chryse outflow channels (Fig. 1A). This geographic nexus is an indicator that cataclysmic flow discharges that initiated within VM^4,14^ were significant contributors to the excavation of large outflow channel areas^4,14^ as well as to the northern plains’ history of sedimentation^3,4,14^ and potential inundation^41–43^. However, our knowledge of the precise VM floods’ discharge mechanisms, sources, and the paleogeography of their precursor paleolakes/seas, remains incomplete and these issues are still subjects of scientific research and dispute (see the supplementary background for likely causes of VM cataclysmic floods).

The SIB is uniquely positioned at the convergence of a vast outflow channel system and the eastern VM canyons (Fig. 1A, blue arrows in Fig. 2A). It is also upstream from Simud and Tiu Valles (Fig. 1B, white arrows in Fig. 2A). This paleo-geographic setting would have limited the flows reaching Simud and Tiu Valles to spillover floods that followed the formation of the inland sea. A fundamental implication of this observational constraint is that extremely voluminous cataclysmic floods likely dominated the flow type discharges from Mars’ most extensive outflow channel system into the northern plains (Fig. 3A, sketches 1–4). This finding provides additional support to the decades-old hypothesis advocating that a northern plains ocean^41–50^ formed during the Late Hesperian due to cataclysmic floods^42,45^ (see the supplementary background for details on the controversy surrounding the Late Hesperian northern ocean hypothesis).

While the SIB’s perimeter lacks discernible shoreline features, potential wave-cut terraces have been identified marking the flanks of numerous mesas in Hydraotes Chaos^51^. The −3,800 m elevation level consistently forms the upper topographic bound to these terraces (Fig. 2E and F). By analogy to the Aral Sea, we propose that marine terrace formation throughout the inland sea shorelines might have been restricted to submerged areas of relatively high inclination (~0.5° to ≥~10°, Fig. S4; Aral Sea supplementary text). In contrast, the terraces would have been absent from regions where high evaporation rates would have driven rapid seafront retreat over shallowly submerged, low inclination coastal plains (~0.01° to ~0.015°, Fig. S4; Aral Sea supplementary text).

The inland sea would have been relatively shallow, typically ~300 m deep, but reaching depths of as much as ~1800 m within Hydraotes Chaos (Fig. S5A). Our simulation of the sea’s thermal stability (supplementary materials on the inland sea’s thermal stability) shows that the sea would have rapidly shrunk as a result of an initial stage characterized by evaporation rates exceeding the rates of surface freezing. This initial stage would have been followed by the formation of a relatively thin ice layer (a few meters thick) covering the sea, which would have rapidly thickened due to high sublimation rates. Also, some of the sea’s volume could have been lost through infiltration into subsurface regolith materials. Within a few thousand years, the sea would have mostly disappeared.

Our model indicates that no significant ice sheets formed, which is consistent with the absence of glacial flow features such as moraines or eskers throughout the basin’s interior landscape. Also, the Hydraotes Chaos mesas and knobs form some of the SIB’s highest relief features, some reaching above the −3,800 m elevation level. However, these promontories lack striations and streamlined or rounded morphologies, which typically form at locations where glaciers overflow prominent topographic features. Instead, wave-cut terraces^51^ flank some of their margins (Figs. 2E and F; S4). The absence of an ice sheet within the SIB reduces the likelihood that glaciers extending from Tiu Valles produced the landscape that we observe at the Pathfinder site^52–54^. Glaciers  however, appear to have played a more prominent role in the geologic histories of other outflow channel regions^6,15,54^.

The estimated SIB sea’s maximum ponding elevation of −3,800 m matches that of the Late Hesperian northern plains ocean’s uppermost paleo-shoreline level described in Rodriguez *et al*.^48^ (Fig. 3B), which they determined based on the lowest distribution of potential tsunami backwash channels. These shoreline stands closely match the elevation mean that Parker *et al*.^43^ estimated for the Late Hesperian northern ocean shoreline (Contact 2, −3760 m ± 560 m). A shared groundwater table through which the two marine bodies reached hydrostatic equilibrium and topographically equalized shorelines best explains their shared elevations. Thus, this topographic correlation comprises an additional piece of evidence in favor of the northern plains ocean hypothesis^41–50^. The matched sea levels are also the highest measured seawater elevations, indicating that equalization must have taken place shortly after the ocean’s emplacement. This observation indicates significant groundwater flow through subsurface conduits^55,56^ (Fig. S5), which connected the basin’s floor and the northern plains through a regionally extensive groundwater table (Fig. 3A, panel 3).

The initial stage in the SIB’s development could have been the result of collapse and subsidence over these conduits (Fig. S5), combined with the effect of large-scale erosion due to episodic cataclysmic floods, which mostly occurred between ~300^29^ and ~100^3^ Ma before the Late Hesperian peak in outflow channel activity. While the SIB’s drainage divide must have changed during the basin’s development and progressive growth, our results indicate that it attained its present position and elevation before the Late Hesperian. To the north, the SIB transitions into broad outflow channel plains (white arrows in Fig. 2), wherein inflowing cataclysmic floods would have spread out, losing velocity and shedding large volumes of their coarser bedload components^6,7^. This process of sedimentation, along with the potential emplacement of tsunami deposits in the region^48^, would have produced a thick build-up of sedimentary deposits, which impounded later flooding events, thereby leading to the formation of the SIB’s sea. Rodriguez *et al*.^57^ showed evidence of an ancient mega-tsunami event in circum-Chryse that was significantly older than those documented in previous work^48^^,^^49^ and which consequently could have contributed to the early phases of sedimentation along the SIB's northern margin. Furthermore, its deposits, which include the location of the Viking 1 Lander landing site, exhibit sufficient run-up distances and elevation gains to have reached the SIB. If the ocean was ice-covered, the hydraulic funneling effect resulting from the wave’s confined propagation between the ice cover and the ocean floor might have produced extremely high-speed mega-tsunami discharges along the frozen ocean’s margins, which could explain the apparent enormity of the mega-tsunami’s onshore propagation^57^. 

The fluvial sedimentology at the Pathfinder site points to an emplacement history due to floods^1^ that were at least an order of magnitude shallower than those reconstructed from remote-sensing data^2^. It also indicates that the latest floods to flow through the Pathfinder site transported a sedimentary bedload that was mostly derived from regional sources^2^. Numerous investigations indicate that the sudden failure of enormous paleolakes^13,22^ and aquifers^3^ within VM likely produced cataclysmic floods in the lower floors of the circum-Chryse outflow channels^4,5^, which mostly occur within the SIB’s extents (see supplementary background on likely causes of VM cataclysmic floods).

**Figure 4:**
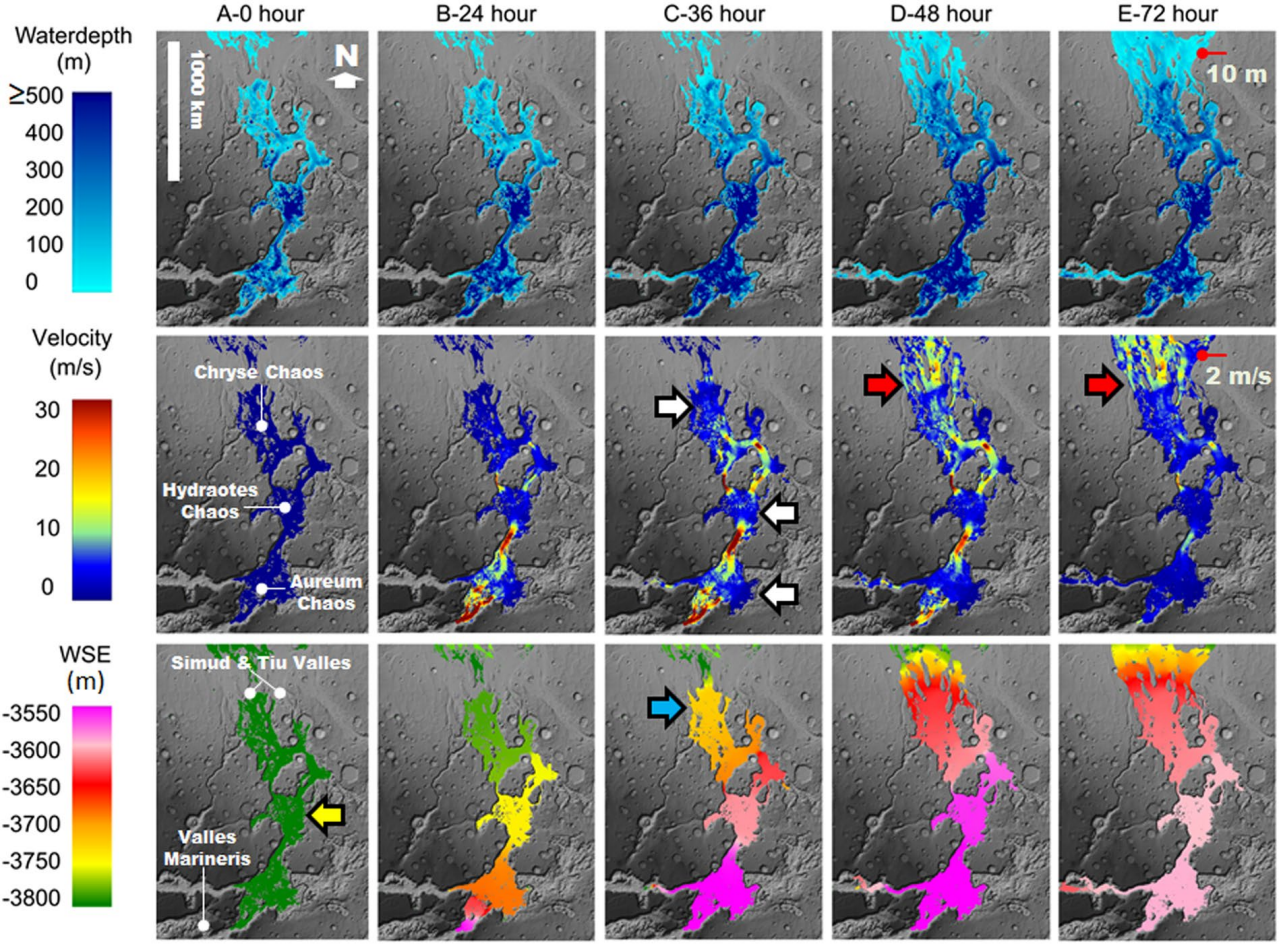
Numerical simulation of a cataclysmic flooding event from eastern VM^13^ into the proposed inland sea. The simulation shows the water depths, velocities, and water surface elevations (WSEs) that developed within the inland sea region and adjoining Simud and Tiu Valles during 72 hours following the cataclysmic flood event. Row A (0 hours) shows an inland sea with a surface elevation at −3,800 m (yellow arrow) covering the SIB. Our modeling assumes that this sea existed at the initiation of the cataclysmic flood event. The simulation shows WSE northwards reductions in the 24, 36, 48, and 72-hour time steps. By the 36-hour mark, relatively low depth floods propagating over the SIB’s northern areas (blue arrow) would have produced marine spillover floods into Simud Valles. The SIB’s spillover discharges would have expanded onto Tiu Valles by the 48- and 72-hour marks. The white arrows identify broad zones of flood deceleration, mostly located within Aureum, Hydraotes, and Chryse Chaos. By the 72-hour mark, the floods reached the Pathfinder landing site. The red arrows show abrupt velocity gains as the floods exited the inland sea as vast marine spillover floods. The red pointers indicate the location of the Pathfinder landing site. Our results indicate that at the landing site, the depth and velocity of the floods were 10 m and 2 m/s, respectively. These depth and velocity values are close matches to those estimated in Smith *et al*.^2^. The simulation was generated using a river flood model (the Martian HEC-RAS 5.0.3). The simulation’s base map is a MOLA-DEM (460 m/pixel) shaded relief. Credit: MOLA Science Team, MSS, JPL, NASA. The subsided plateaus in the Aureum Chaos region show no evidence of overflow erosion, indicating that subsidence was subsequent to the sea’s formation (Fig. S7)^55,62^. Consequently, we corrected the DEM at the contact between this chaotic terrain and the margin of the inland sea by creating a flow barrier (Fig. S7).

We modeled the abrupt release of ~1.2^14^ m^3^ of lake water from VM into the inland sea during a total period of 72 hours (Fig. 4; see supplementary methodology for flood modeling approach). The sea’s formation would have submerged the SIB’s reversed gradient channel-floor areas leading to lower Simud and Tiu Valles^21–25^, thereby providing a constant elevation surface between eastern VM and these outflow channels (yellow arrow in Fig. 4). As a consequence, the presence of the inland sea would have permitted the long-distance propagation of low depth floods in southern circum-Chryse.

Our simulation shows an initial peak discharge, after a 24-hour time interval, at 1.0^9^ m^3^/s, reaching depths and flow velocities of ~250 m and ~30 m/s, respectively. These depth and velocity values fall within the ranges estimated to have led to the formation of outflow channel multi-kilometer scale features observed in remote-sensing data^10^. The simulation shows that the cataclysmic flood significantly raised the water surface elevations (WSEs) above the −3,800 m elevation level within the inland sea’s extents. However, consistent northwards WSE reductions, as shown in the 24, 36, 48, and 72-hour time steps, indicate that the inland sea’s presence could have effectively attenuated the depths of inflowing cataclysmic floods. By the 36-hour mark, relatively low depth floods would have initiated marine spillover discharges into the Simud Valles (e.g., blue arrow in Fig. 4, flood depth to the −3,800 elevation level). These spillover flows have subsequently expanded eastwards into Tiu Valles during the 48 and 72-hour marks.

The 48 and 72-hour marks show that the floods propagating over the inland sea gained velocity (~10 m/s to ~20 m/s) at the spillover boundary into Simud Valles (red arrows in Fig. 4). Thus, the spillover floods would have entrained large (multiple meters in size^11^) clasts derived from the inland sea’s northern margin and the outflow channel floors. The acquisition of this sedimentary load explains the fact that the proposed fluvial sediments observed at the Pathfinder site were found to have been likely derived from nearby source areas^2^. By the 72-hour mark, marine spillover floods ~10 m in depth and moving at ~2 m/s would have reached the Pathfinder site. The simulation ends at the 72-hour mark due to an agreement between these hydraulic parameters and those obtained from *in-situ* observations^2^. Our results reconcile the apparent enormity of the cataclysmic regional flooding^10^, as inferred from orbital remote-sensing data, with the much less intense fluvial processes documented from *in-situ* observations at the Pathfinder site^2^.

The simulation reveals abrupt flood velocity losses (~30 m/s to <10 m/s) during flow through Aureum, Hydraotes, and Chryse Chaos (white arrows in Fig. 4). The resulting reduced sediment-transport capacity would have led to the dislodging of coarse bedload materials (probably mostly clasts larger than a meter^11^) into these regions. Moreover, the modeled cataclysmic flood discharge would have significantly increased the inland sea’s depth (Fig. 4), resulting in powerful flood currents with typical underlying seawater depths of several hundred meters. Basal intermixing-induced deceleration, perhaps enhanced along the sea level interface, might have further increased the shedding of bedload materials.

The flooding history of the SIB’s region was likely episodic^3,4,6,7^. Variations in the depths and velocities of inflowing cataclysmic floods, as well as in the resulting inland sea paleogeographic configurations, would have likely affected the hydraulic parameters of the floods reaching the Pathfinder site. However, a fundamental interpretation of our modeling is that the formation of an extensive sea would have permitted the floods to reachbeyond the inundated reversed channel floor areas, despite the sepotential variations.

Extensive Late Noachian marine deposits, emplaced when an Earth-like surface hydrologic system is thought to have existed on Mars, likely cover the Eridania basin^27,58^. In contrast, while the Late Hesperian northern plains ocean hypothesis^41–47^ has gained support in recent years^48–50^, to date the ocean’s floor materials and features have not been unambiguously identified^3,14^. A fundamental obstacle towards the recognition of these materials is that detailed stratigraphic reconstructions of the northern plains^3,14^ indicate their most probable occurrence as mostly buried beneath the Vastitas Borealis Formation (VBF)^14,59^ (Fig. S6), a geologic unit identified as the Late Hesperian lowland unit on the latest geologic map of Mars^3^. The VBF covers most of the proposed paleooceanic basin, and it is likely made up of the frozen ocean’s geologic residue^45,59,60^. In contrast to the northern plains paleoocean basin, our numerical modeling and geologic observations indicate that the proposed inland sea, and its frozen residue materials, disappeared within approximately a thousand years (see supplementary materials on the inland sea’s thermal stability). Consequently, the basin floor materials might comprise the most extensive Late Hesperian marine sedimentary deposit currently existing at the Martian surface.

Enormous groundwater-fed lakes and vast aquifers are thought to have comprised the sources of the floods that formed the inland sea. These aquifers might have formed during the Middle and Late Noachian^56^ [~3.94 to ~3.71 Ga^3^ as well as during the Hesperian Period^45^ [~3.71 to ~3.37 Ga^3^ due to the gradual assimilation into the subsurface of surface and atmospheric water. Consequently, some of the aquifers possibly existed for ~540 Ma. Furthermore, it is possible that these aquifers were geothermally heated^59^ and could have had contact with hydrothermal systems, creating environments with potential sources of liquid water, nutrients and energy for putative organisms. Thus, we suggest that this inland sea’s sedimentary record is a prime target for missions to search for evidence of ancient life within early Mars aquifers.

## Supplementary information

Supplements
