# Supplementary material for: The 1997 Mars Pathfinder Spacecraft Landing Site: Spillover Deposits from an Early Mars Inland Sea
Source: Sci Rep. 2019 Feb 25;9:4045. doi: 10.1038/s41598-019-39632-1 (PMC6401135; doi:10.1038/s41598-019-39632-1)
Supplement: Supplementary file 1 — Supplements [file 41598_2019_39632_MOESM1_ESM.docx]

**Supplementary Materials to:**

**The 1997 Mars Pathfinder Spacecraft Landing Site: Spillover Deposits from an Early Mars Inland Sea**

J. A. P. Rodriguez^1*^, V. R. Baker^2^, T. Liu^2^, M. Zarroca^3^, B. Travis^1^, T. Hui^2^, G. Komatsu^4^, D. C. Berman^1^, R. Linares^3^, M. Sykes^1^, M. E. Banks^1,5^, and J. S. Kargel^1^ (see author contribution statement for details).

*Correspondence to alexis@psi.edu

*^1^Planetary Science Institute, 1700 East Fort Lowell Road, Suite 106, Tucson, AZ 85719-2395, USA.*

*^2^Department of Hydrology & Atmospheric Sciences, University of Arizona, Tucson, AZ 85721, USA.*

*^3^External Geodynamics and Hydrogeology Group, Department of Geology, Autonomous University of Barcelona, 08193 Bellaterra, Barcelona, Spain.*

*^4^International Research School of Planetary Sciences, Università D'Annunzio, Viale Pindaro 42, 65127 Pescara, Italy.*

*^5^NASA Goddard Space Flight Center, Goddard MD 20771 USA.*

**Table of contents**

**Supplementary Figures 3**

**Supplementary Methods 12**

**Supplementary Background 16**

**The Aral Sea as an Analog to the Inland Sea Supplement 24**

**The Inland Sea’s Thermal Stability Supplement 28**

**Supplementary Figures**

**
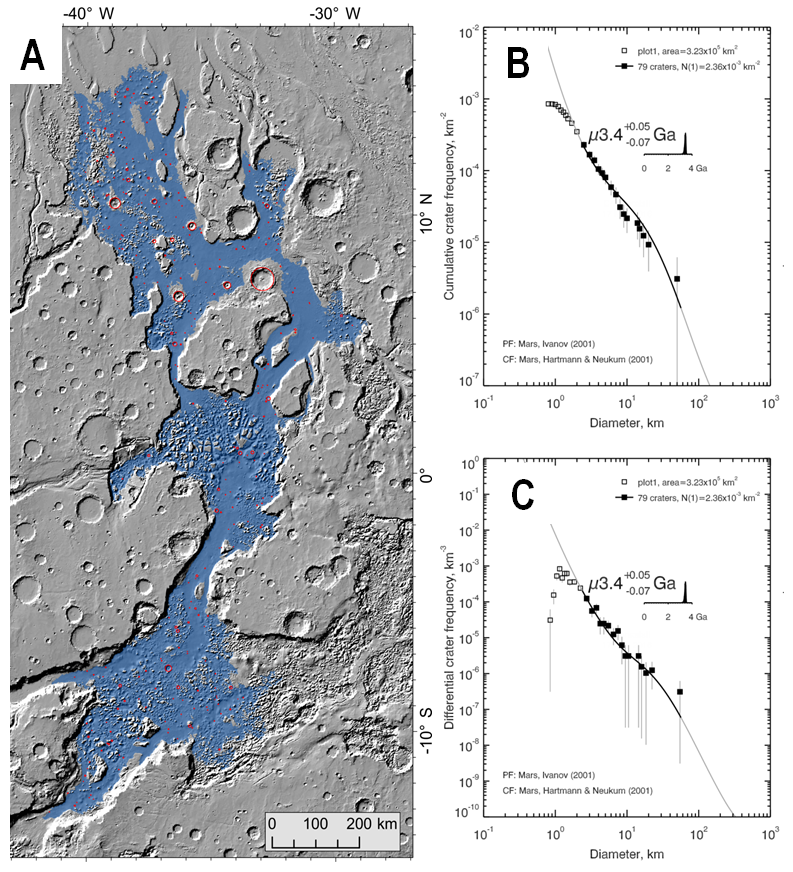
**

**Figure S1 (A)** Map showing the counting area (blue) and mapped craters (red circles). The background image is a Mars Orbiter Laser Altimeter (MOLA) digital elevation model (460 m/pixel, credit: MOLA Science Team, MSS, JPL, NASA) derived hillshade in sinusoidal projection centered at 325°E. **(B, C)** We obtained a crater-based model age of ~3.4 Ga from craters exposed on the basin’s floor. Panels B and C are reverse cumulative and differential crater size-frequency plots, respectively.

**
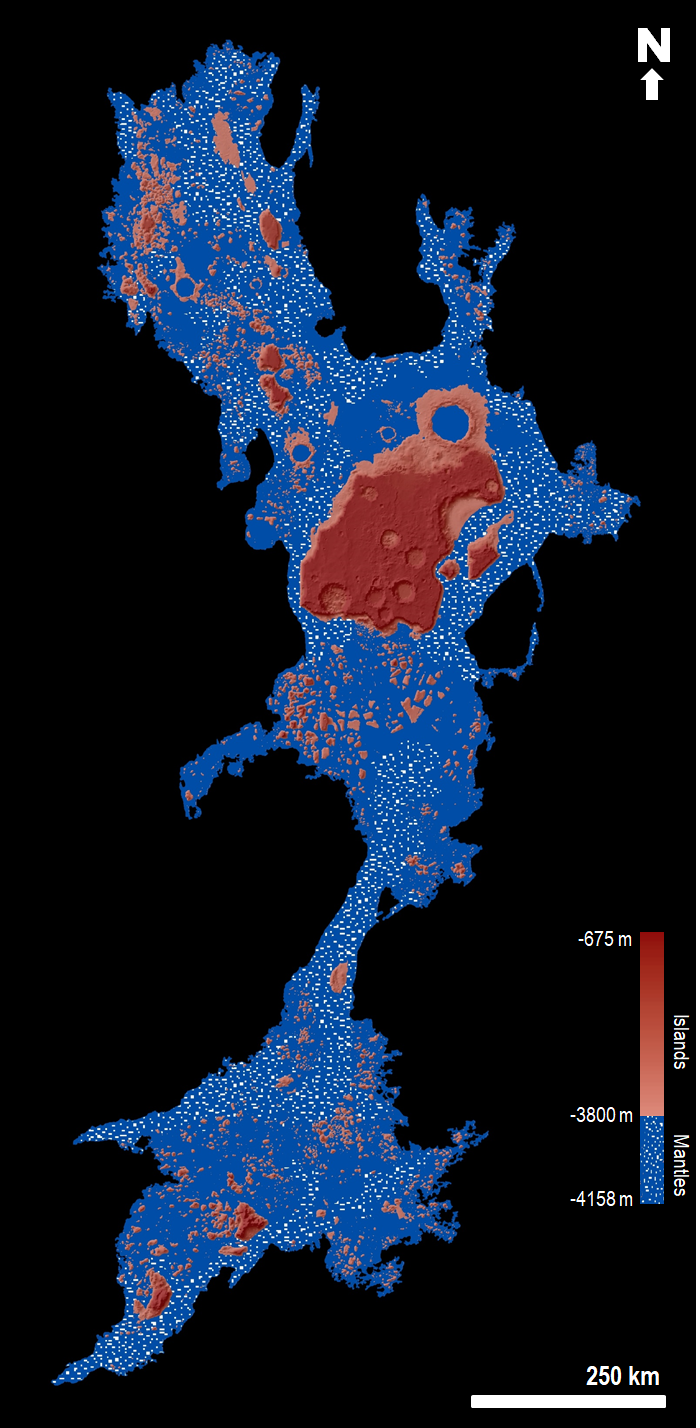
**

**Figure S2** View of the SIB’s surface showing a -3,800 m elevation topographic mask (blue), interior “islands” comprised of promontories that stand above this elevation (red), and the full extent of the mapped sedimentary unit (white specks, Fig. 2A). Notice that these materials are topographically bounded below the -3,800 m elevation. We used a MOLA digital elevation model (460 m/pixel) to produce this figure. Credit: MOLA Science Team, MSS, JPL, NASA.


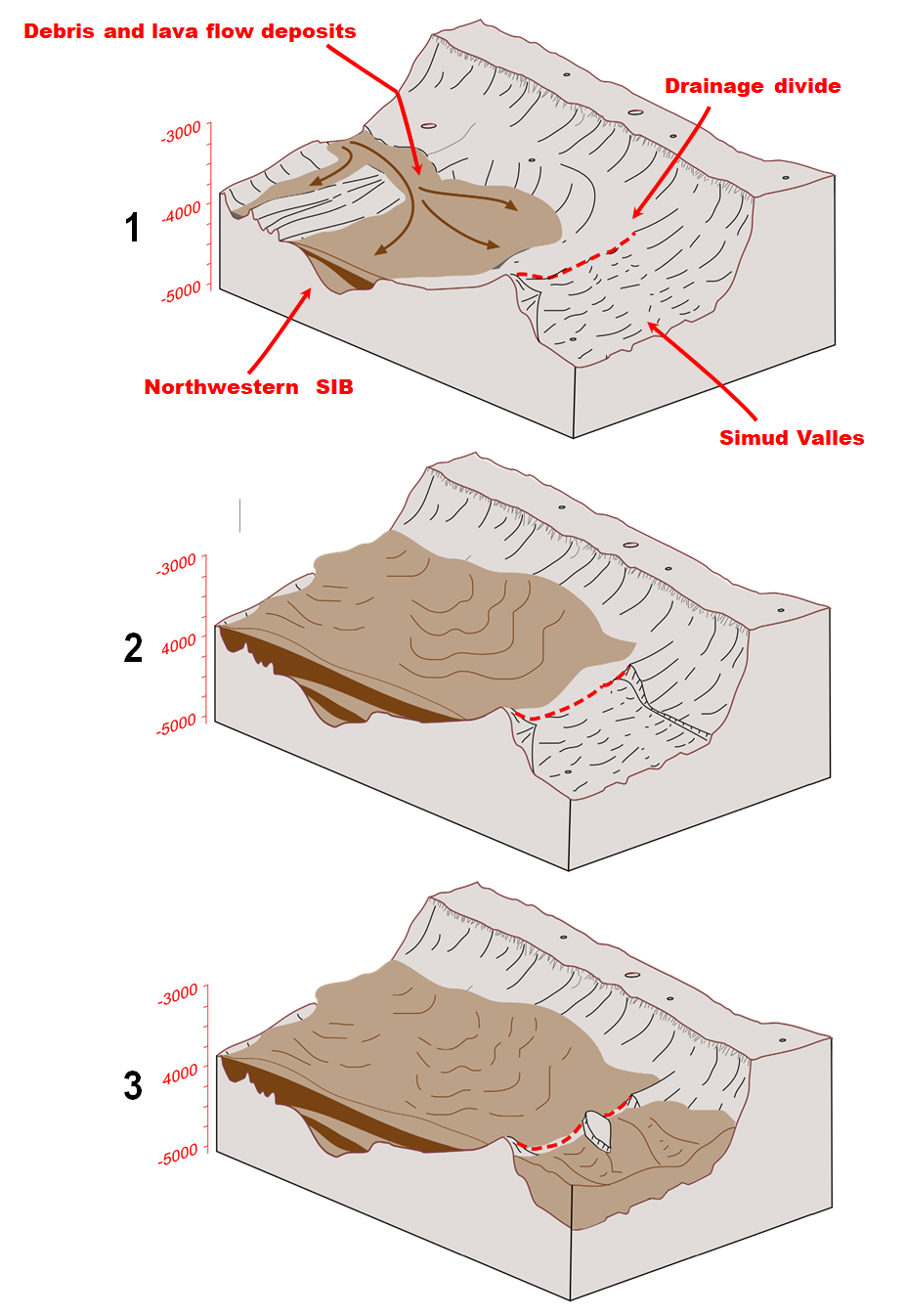


**Figure S3** Perspective sketches showing the SIB’s northwestern part. The region’s context and location (Fig. 3B) are those of Fig. 3A. The sketches depict the basin’s progressive burial due to a history of outflow channel activity dominated by lava and debris flows.


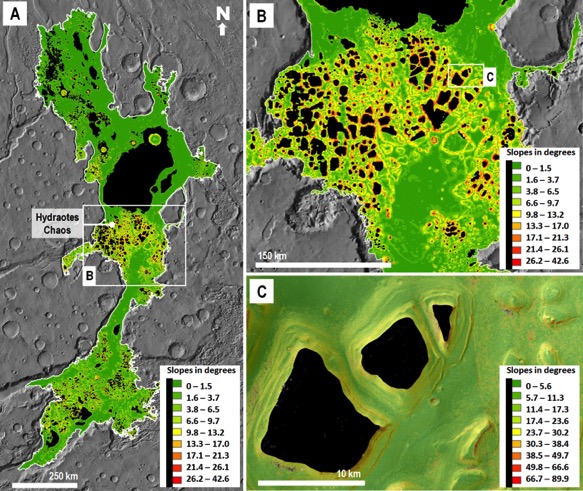


**Figure S4 (A)** MOLA-generated slope map centered at 1°18'N, 34°10' W (460 m/pixel, credit: MOLA Science Team, MSS, JPL, NASA) of the SIB interior floors below the -3,800 m elevation level. We masked in black the SIB’s interior surfaces located above this elevation. The submerged terrain slopes that extend from the basin’s margins are mostly below ~5**°**. **(B)** In contrast, those that flank the scarps of numerous mesas within Hydraotes Chaos are significantly steeper, typically ranging from ~13° to ~17°. **(C)** Close-up view of some of Hydraotes Chaos interior mesas using CTX topography over a CTX image mosaic (~6 m/pixel; credit: NASA/JPL. The license terms can be found at pds-imaging.jpl.nasa.gov/portal/mro_mission.html). The black mask covers the portions of the mesas above the -3,800 m elevation level. The increased topographic resolution in the view reveals hypothesized formerly submerged mesa flanks reaching inclinations of ~90°.

**
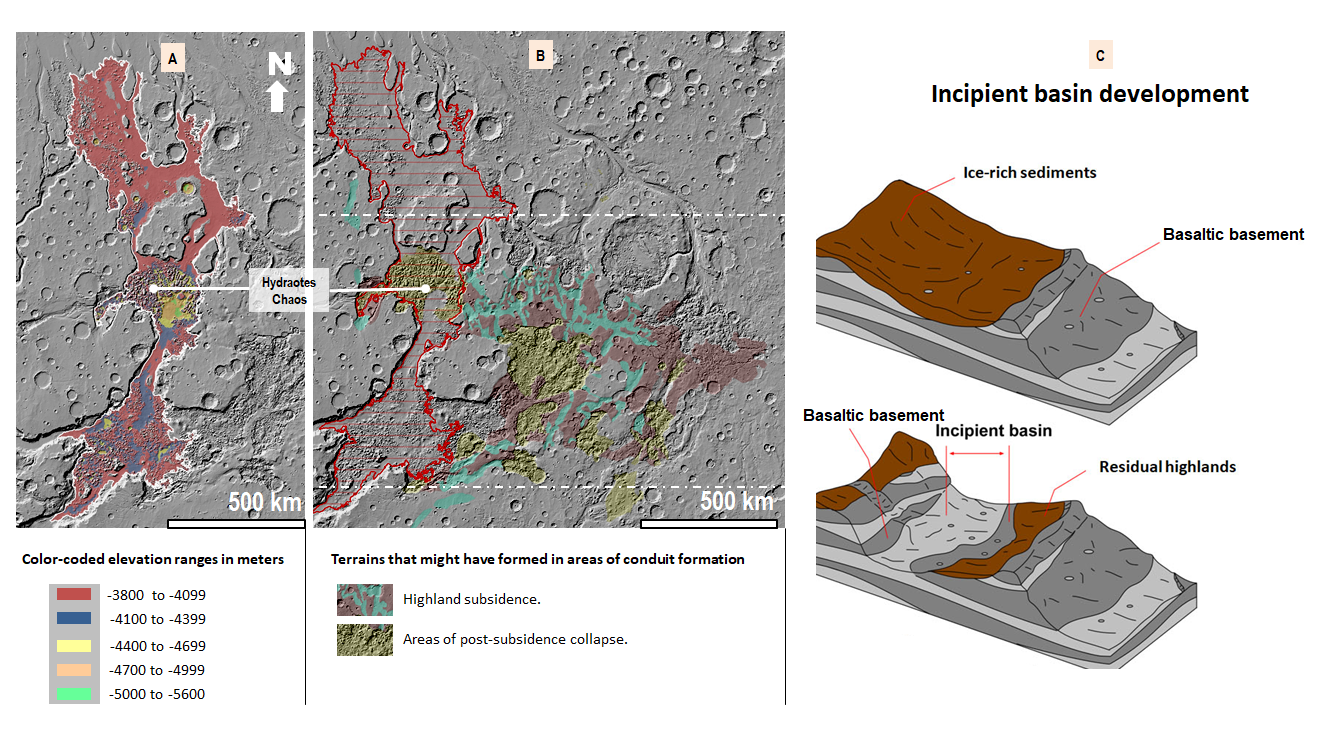
**

**
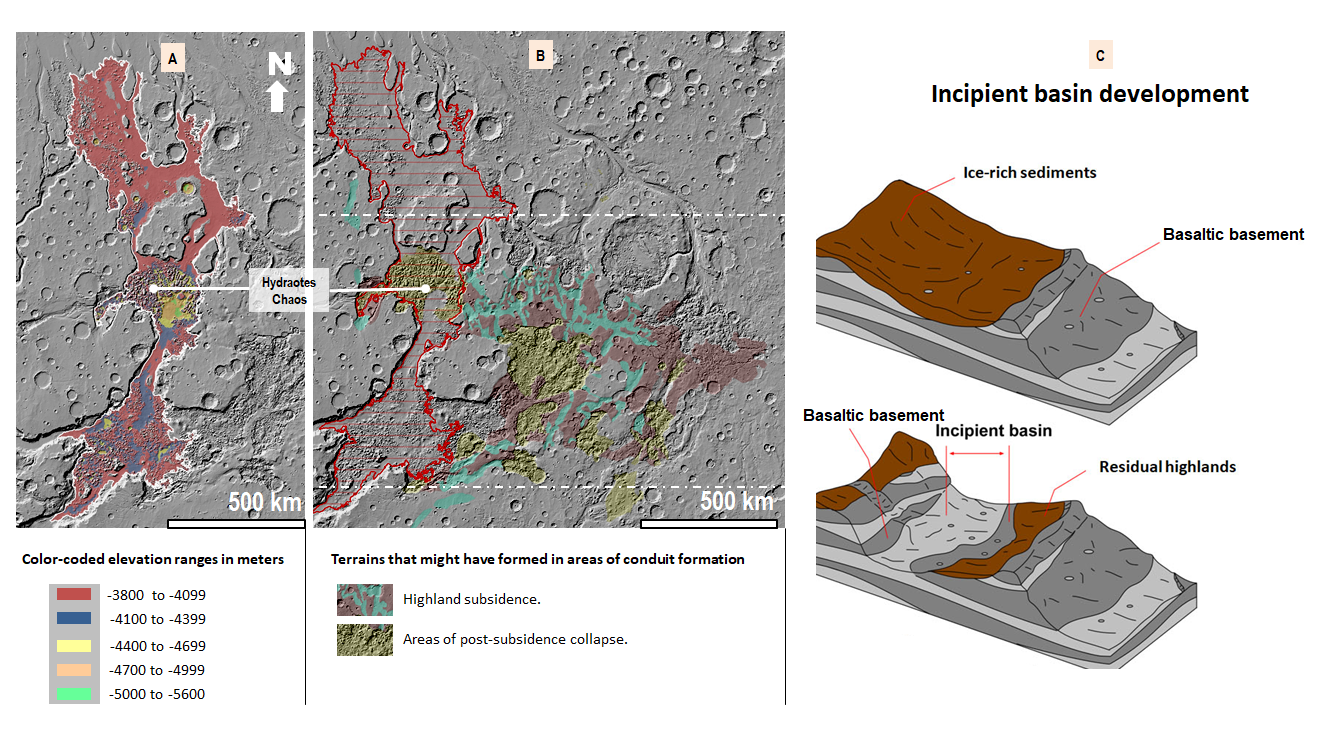
**

**Figure S5 (A)** MOLA-generated view centered at 1°18'N, 34°10' W (460 m/pixel; credit: MOLA Science Team, MSS, JPL, NASA) showing the SIB’s interior depth distribution below an elevation of -3,800 m. While the basin’s floor generally lies at depths ranging between ~300 and ~400 m, its deepest part, located in Hydraotes Chaos, reaches a maximum depth of ~1,800 m. **(B)** Morphologic map over a MOLA-generated shaded relief base (460 m/pixel; credit: MOLA Science Team, MSS, JPL, NASA). The view shows the distribution of highlands areas affected by subsidence and post-subsidence collapse in southern circum-Chryse, based on mapping by Rodriguez *et al*.^1^. Subsidence likely occurred in response to the collapse of widespread subterranean conduits from which some of the catastrophic floods that formed the outflow channels emerged during the Late Hesperian^1^. The two dashed white lines mark the approximate latitudinal distribution of these terrains, and the red lines demark the SIB’s margins and its interior regions. Notice that Hydraotes Chaos forms the deepest of the subsided terrains, implying the former presence of deep-seated conduits in the region. These conduits might have contributed to equalizing the maximum shoreline levels within the inland sea and the northern plains ocean (Sketch 3 in Fig. 3A, Fig. 3B). **(C)** Conduit formation likely occurred within a Middle Noachian ice-rich sedimentary wedge^1-3^ that was emplaced on top of an ancient basaltic basement^1^. The two perspective sketches illustrate early regional subsidence and collapse within the upper crust-forming sedimentary wedge as a mechanism to initiate the basin’s incipient development.

**
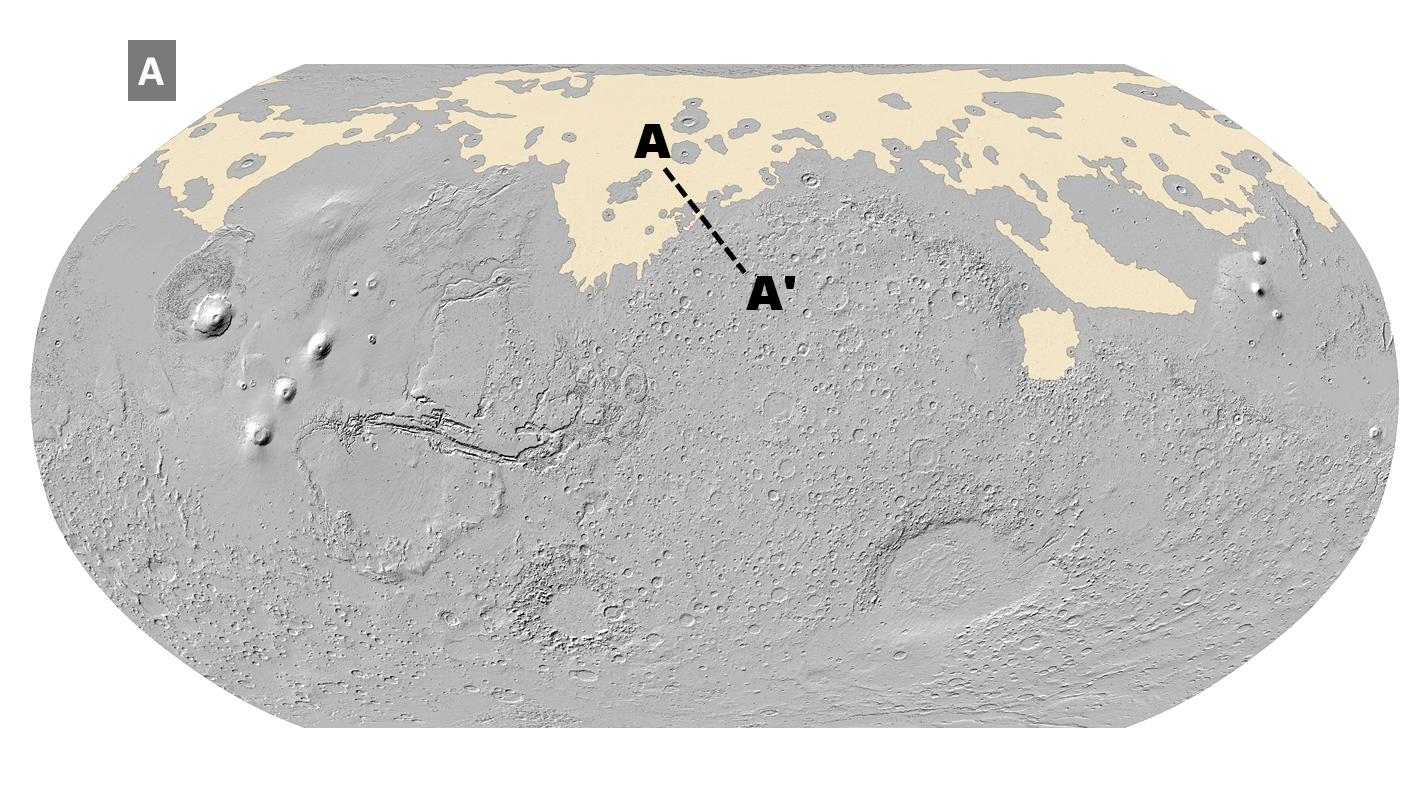
**

**
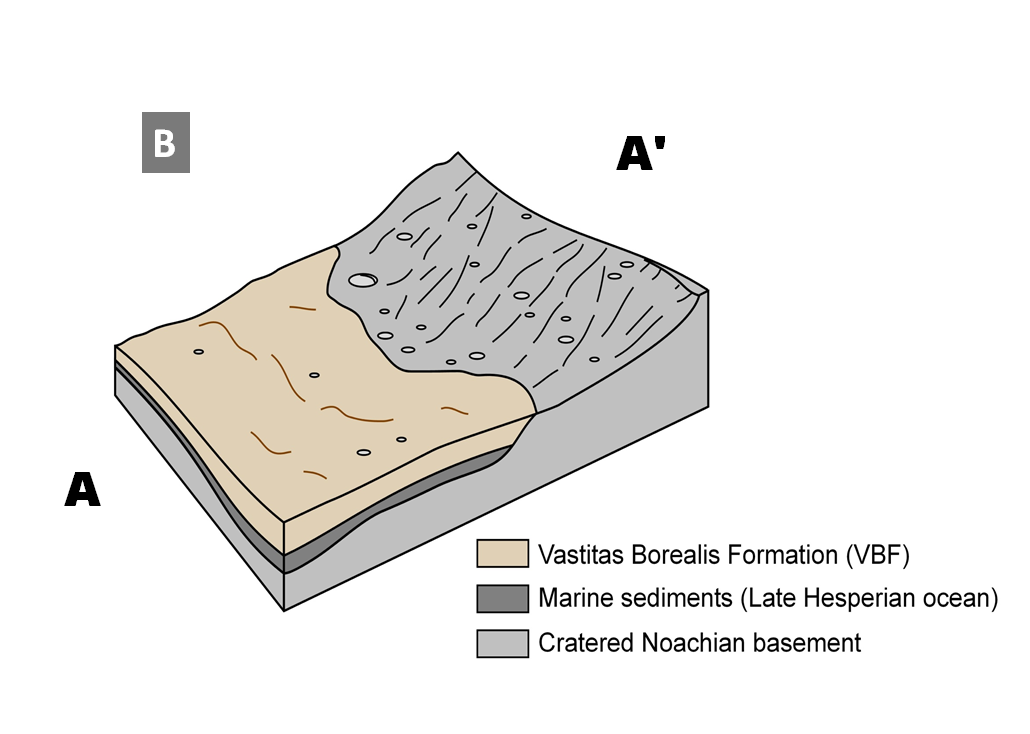
**

**Figure S6 (A)** View showing the distribution of the Late Hesperian lowland unit^4^ (pastel color, previously mapped and generally known as the Vastitas Borealis Formation, VBF^5^) extracted from the latest global geologic map of Mars^4^. The dashed black line marked A to A' marks the location of the elevation profile in panel (B), which traces the highland-lowland boundary between the VBF and the adjoining Arabia Terra highlands. The image base is a MOLA-generated shaded relief map (460 m/pixel; credit: MOLA Science Team, MSS, JPL, NASA). **(B)** Regional stratigraphic reconstruction along the elevation profile A to A' extracted from a MOLA digital elevation model (460 m/pixel; credit: MOLA Science Team, MSS, JPL, NASA). The reconstruction, which is partly based on existing geologic mapping^4,5^, positions the proposed Late Hesperian marine floor sediments above an older Noachian basement and below the VBF.

**
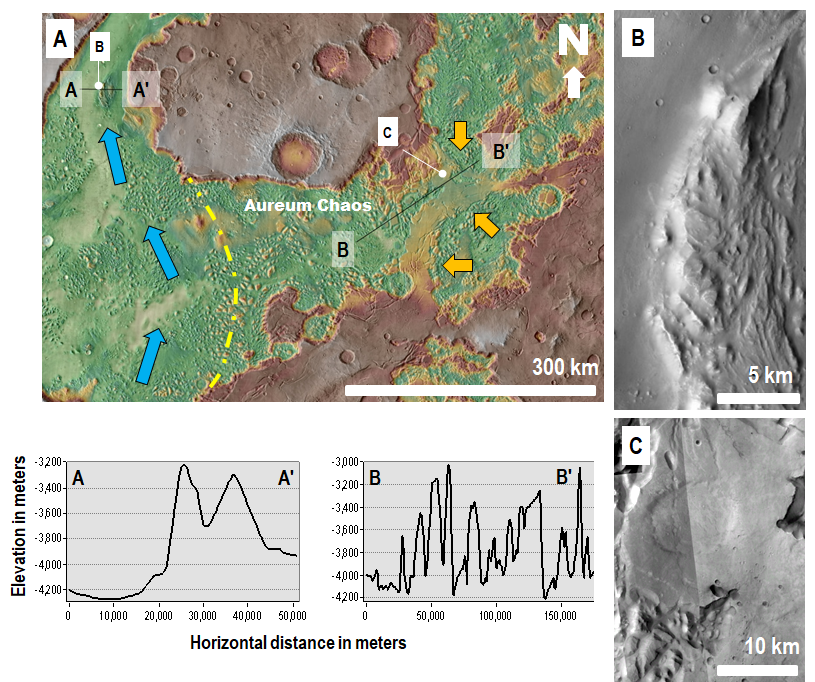
**

**Figure S7 (A)** View of eastern Aureum Chaos centered at 7° 9'14"S, 31° 2'44"W (MOLA-generated digital elevation model (460 m/pixel; credit: MOLA Science Team, MSS, JPL, NASA) overlying a Thermal Emission Imaging System (THEMIS) Day IR (infrared) 100 m Global Mosaic. (Credit: Christensen, P.R., Gorelick, N.S., Mehall, G.L., & Murray, K.C. *THEMIS Public Data Releases*, Planetary Data System node, Arizona State University, <http://themis-data.asu.edu>. Date of access: 12/21/2018.) To conduct the numerical simulation shown in Fig. 4, we corrected the effect of subsidence in eastern Aureum Chaos by creating a topographic barrier to the simulated cataclysmic flood from eastern Valles Marineris (VM). The blue arrows show the flood’s approximate pathway in the region. The dashed yellow line traces the barrier, which has an elevation set at -1,000 m. Eastern Aureum Chaos likely experienced widespread subsidence^2, 3^ after the end of major cataclysmic flood discharges from VM. This proposed sequence of events is consistent with the fact that an outflow channel interior mesa located downstream from VM has a prominently scoured surface **(B, elevation profile A to A')**. While the mesa shares similar elevation and relief ranges to those of the subsided highland areas adjoining the SIB’s southern margin (orange arrows), the latter lack evidence of erosional overflow **(C, elevation profile B to B')**. The ends of the white pointers in panel A indicate the center location of panels B and C. Panels B and C are parts of a regional CTX (~6 m/pixel) mosaic. The license terms can be found at pds-imaging.jpl.nasa.gov/portal/mro_mission.html.

* We produced the mosaic areas and maps in the supplementary figures using [Esri](http://en.wikipedia.org/wiki/Esri)'s ArcGIS^®^ 10.3 software (<http://www.esri.com/software/arcgis>).

**References cited in the supplementary figures**

1 Rodriguez, J. A. P. *et al.* Martian outflow channels: How did their source aquifers form, and why did they drain so rapidly? *Scientific Reports* **5**, Article number: 13404 doi:10.1038/srep13404 (2015).

2 Rodriguez, J. A. P. *et al.* Outflow channel sources, reactivation, and chaos formation, Xanthe Terra, Mars. *Icarus* **175**, 36-57 (2005).

3 Rodriguez, J. A. P. *et al.* Control of impact crater fracture systems on subsurface hydrology, ground subsidence, and collapse, Mars. *J. Geophys. Res.* **110**, DOI: 10.1029/2004JE002365 (2005).

4 Tanaka, K. L. *et al.* Geologic map of Mars. U.S. Geological Survey Scientific Investigations Map 3292, scale 1:20,000,000, <http://pubs.usgs.gov/sim/3292/> (2014) Date of access: 03/14/2018.

5 Tanaka, K. L., Skinner, J. A. & Hare, T. M. Geologic map of the northern plains of Mars. U.S. Geological Survey Scientific Investigations Map 2888, scale 1:15,000,000 (1 mm = 15 km) at 90° N and 1:7,500,000 at 0° N, <http://pubs.usgs.gov/sim/2005/2888/> (2005) Date of access: 03/14/2018.

**Supplementary Methods**

**Mapping Approach**

The figure maps were produced using Esri's ArcGIS^®^ 10.3 software (<http://www.esri.com/software/arcgis>). We identified and defined the extent of the Simud Interior Basin (SIB) using part of a Mars Orbiter Laser Altimeter (MOLA) global digital elevation model (DEM, ~460 m/pixel horizontal; ~1 m vertical resolution). We identified and mapped the distribution of the SIB’s interior smooth deposits utilizing Mars Reconnaissance Orbiter (MRO) Context Camera (CTX, ~6 m/pixel) visible light image mosaics as well as Mars Odyssey Thermal Emission Imaging System (THEMIS) daytime (~100 m/pixel) and nighttime (~100 m/pixel) infrared (IR) light image mosaics in combination with MOLA topography. Also, we used CTX-derived topography to investigate the elevations of terraces bounding the margins of numerous mesas within Hydraotes Chaos. The CTX DEMs were constructed from overlapping CTX images G05_020047_1809 and G19_025506_1809 (left) and F09_039128_1799 and F10_039761_1798 (right) using the MarsSI automated pipeline (<https://emars.univ-lyon1.fr/MarsSI/>).

**Volumetric Characterizations**

The calculation of the SIB’s interior volume was performed using MOLA topography. We used the surface volume tool in GIS, inputting an extracted topography from within the basin’s boundaries and setting the plane of reference to -3,800 m elevation.

**Surface Age Determinations**

To estimate the surface age of the proposed inland sea basin we first selected the -3,800 m contour line from the MOLA DEM as its inferred outline. Any higher standing terrain was then removed from this region resulting in an areal size of 323,308 km^2^. All craters that formed in the region ≤3,800 m in elevation were counted and measured using the ArcGIS add-in *CraterTools*^1^. Some craters located at slightly higher elevations, e.g., Mojave crater, were included in this analysis as their ejecta superimpose the low-lying terrain. The crater size-frequency distribution was analyzed using *Craterstats*^2,3^ by applying the chronology and production functions of Hartmann and Neukum^4^ and Ivanov^5^, respectively. The approximate crater-based model age is 3.4 Ga (1σ: +0.05 Ga, -0.07 Ga). The crater size-frequency distribution does not perfectly match the production function, which is likely the result of differential resurfacing at different sites within the basin. Therefore, the approximate model age of ~3.4 Ga should be regarded as the minimum age.

**Flood Modeling Approach**

We modeled the hydraulics of the mega-flooding released from an enormous paleolake contained within Valles Marineris using HEC-RAS 2-D. The Hydrologic Engineering Center's River Analysis System (HEC-RAS) software allows the user to perform one-dimensional (1D) steady and 1D and two-dimensional (2D) unsteady flow river hydraulic calculations (www.hec.usace.army.mil/software/hec-ras/documentation/HEC-RAS 5.0 2D Modeling Users Manual.pdf). This model simulated a 2D unsteady flow by solving full Saint-Venant equations with an implicit finite volume solution algorithm on both structured and unstructured meshes^6^. This full momentum-based equations set can more accurately capture the flow dynamics in the following situations: (1) highly dynamic flood waves generated from quick dam failure; (2) mixed flow regimes and hydraulic jumps when floods propagated through channel constrictions; and (3) detailed velocities, water depth, and other hydraulic parameters within locations and times of interest^6^. These capabilities enable us to accurately and efficiently describe flow strength features^6^. The gravitational acceleration of the Martian version (HEC-RAS (v5.0.3)) was set to 3.711 m/s^2^. We used a regional MOLA DEM (~460 m/pixel horizontal; ~1 m vertical resolution) as the base layer for our simulations. The computational mesh was generated from the DEM with a 2000-meter grid space for most regions. However, hydrologic characterizations at the Pathfinder landing site were determined using finer resolution grids. We assigned a 0.035 Manning coefficient value for the whole domain^7^.


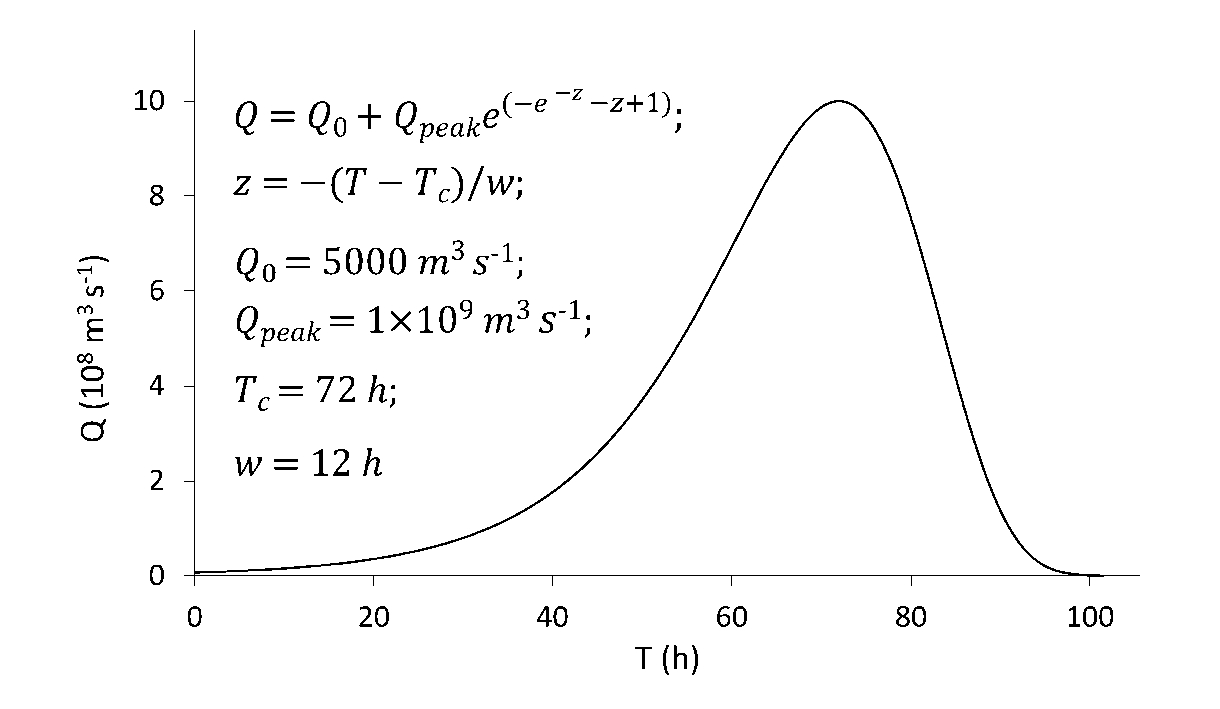
Determining the hydrography of outburst megafloods is challenging because of their uncertain trigger mechanisms and the complexities associated with the modeling of the dam-breach process. The flow hydrograph used in this study is, therefore, represented by a generalized extreme value (GEV) distribution function (below). Note that the hydrograph has a broad rising limb and sharp falling limb with a 1E9 m^3^/s peak discharge and a release of ~1.2E14 m^3^ of lake water within 100 h. The simulation used a normal water depth boundary condition, which allowed for the floodwaters to extend beyond the domain region.

Lake outflow hydrograph used in this study with a 1E9 m3/s peak flow and 100 h duration.

References cited in supplementary methods

1 Kneissl, T., *et al.* Map-projection-independent crater size-frequency determination in GIS environments–New software tool for ArcGIS. *Planet Space Sci.* **59**, 1243-1254 (2011).

2 Michael G. G. & G. Neukum. Planetary surface dating from crater size-frequency distribution measurements: Partial resurfacing events and statistical age uncertainty. *Earth Planet. Sci. Lett.* **294**, 223-229 (2010).

3 Michael G. G. *et al.* Planetary surface dating from crater size-frequency distributions: Poisson timing analysis. *Icarus* **277**, 279-285 (2016).

4 Hartmann, W. K. & Neukum, G. Cratering chronology and the evolution of Mars. *Space Sci. Rev.* **96**, 165-194 (2001).

5 Ivanov, B. A. Mars/Moon cratering rate ratio estimates. *Space Sci. Rev*. **96**, 87-104 (2001).

6 Brunner, G. HEC-RAS River Analysis System 2D Modeling User’s Manual. Version 5.0 (2006).

7 Burr, D. & McEwen, A. Recent aqueous floods from the Cerberus Fossae, Mars. *Geophysical Research Letter* **29**, 13-1—13-4 (2002).

**Supplementary Background**

During the Late Hesperian Period, Mars experienced one of the most dramatic episodes in its hydrologic history^1-3^. Enormous cataclysmic floods proposed to have reached the northern plains via the Chryse outflow channels were likely sufficiently voluminous^4, 5^ to produce a northern plains ocean^4-12^. The ocean hypothesis remains controversial. A fundamental observation in favor of the Late Hesperian ocean^4, 5^ is the fact that the northern plains are covered by a vast ice-rich deposit ^9, 12, 13^ with margins that overlap, and thus postdate the floods of the Chryse outflow channels’ lower reaches^3, 13^. Other lines of consistency include the presence of widespread, large-scale polygons of likely submarine origin^11, 12^.

However, since this hypothesis was first put forward by Parker *et al*.^4, 5^, the idea remained shrouded in controversy due in part to two apparent inconsistencies. First, instead of Earth-like shoreline features such as wave-cut terraces^14^, remote-sensing analyses showed that lobate deposits bound the proposed paleo-ocean basin^13, 15^. Secondly, instead of conforming to the ocean’s surface constant elevation, the proposed shoreline features appear distributed throughout wide elevation ranges^7^.

The discovery of potential tsunami deposits, as discussed in recent articles by Rodriguez *et al*.^16^ and Costard *et al*.^17^, offer a simple solution in which mega-tsunami deposits likely dominate the preserved geologic record of early Mars coastal terrains. Tsunamis typically result in the emplacement of deposits with upper boundaries distributed above the shorelines’ equipotential topographic position^18, 19^. Mapping of these deposits by Rodriguez *et al*.^16^ throughout the highland-lowland boundary areas of circum-Chryse and northwestern Arabia Terra reveals typical widths and lengths reaching several hundred kilometers as well as characteristic relief gains of a few hundred meters. Furthermore, deformation caused by the emplacement of Tharsis likely contributed to some of the large variations in shoreline topography^20^.

Decades of investigations indicate that huge discharges from Valles Marineris (VM) comprised significant sources to the floods that could have formed the proposed Late Hesperian ocean^1-3, 6, 8^. Their generating mechanisms could have involved the structural failure of giant groundwater-fed paleolakes/seas^2, 21-24^ as well as the evacuation and collapse of enormous highland aquifers^2, 3, 25, 26^.

Evidence of cataclysmic outflows from the proposed multi-kilometer deep paleolakes/seas includes the orientation and topographic distribution of scour marks in the walls of the easternmost chasmata^22, 24^ as well as extensive deposits with lobate fronts extending from breached tectono-volcanic basins^25^. Evidence that the paleolakes/seas were stable for significant time lengths includes the presence of thick (a kilometer or more) Interior Layered Deposits (ILDs)^21-25, 27-32^.

The ILD contain abundant hydrated sulfates and hydroxyl minerals that could have formed due to then episodic mineral precipitation within the ponded bodies of water^30, 31^. However, several hypotheses have been proposed to explain the ILD formation. These include dry-land spring mound discharges^33^, the aqueous saturation of aeolian mantles^34^, the trapping of dust and sulfur aerosols within relict glacial ice deposits^35, 36^, and sub-ice volcanism^37^.

No doubt, the widespread resurfacing within VM by tectonism, landslides, glaciation, and volcanism^1-3^ complicates our current knowledge of the precise paleo-geography of potential VM interior paleolakes/seas. Hence, there is valid room for controversy. Our premise, however, is that in addition to these other processes, the more obvious and perhaps more oft-considered hypothesis is more likely correct: that there is a close relationship between the VM ILD deposits (formed by standing water) and the Chryse outflow channels (formed by outbursts of that same water). Consequently, we proceed on the premise that there were deep paleo-lakes in the VM and immense floods of water that issued from them.

Decades of research indicate that the volumes and discharge rates of the fluids issued from VM during the Late Hesperian had to be sufficiently high to excavate the outflow channels and to cover the northern plains with sediments^2, 3^. Furthermore, this phase of outflow channel activity likely produced a northern plains ocean^6-12^, as has been long hypothesized and supported by some recent discoveries^10-12^. However, we note that the volume estimates from the proposed areas of water evacuation within VM do not necessarily provide an upper limit to the volumes of water that could have reached the northern plains. The potential discrepancy is because surface and subsurface bodies of water within VM are thought to have formed due to the episodic drainage into VM of vast aquifers that existed within the Tharsis rise^38-41^, an enormous volcanic bulge which bounds the western-most reaches of this canyon system.

**References cited in the supplementary background**

1 Scott, D. H. & Tanaka, K. L. Geologic map of the western equatorial region of Mars, U.S. Geological Survey Scientific Investigations Map I-1802-A, scale 1:15,000,000, ISBN: 978-0-607-89775-3, <http://pubs.er.usgs.gov/publication/i1802A> (1986) Date of access: 0707/2015.

2 Rotto, S. & Tanaka, K. L. Geologic/geomorphic map of the Chryse Planitia Region of Mars Geologic map of the western equatorial region of Mars, U.S. Geological Survey Scientific Investigations Map, I-2441-A, scale 1: 5,000,000, <http://pubs.er.usgs.gov/publication/i2441> (1995) Date of access: 03/14/2018.

3 Tanaka, K. L. *et al.* Geologic map of Mars. U.S. Geological Survey Scientific Investigations Map 3292, scale 1:20,000,000, <http://pubs.usgs.gov/sim/3292/> (2014) Date of access: 03/14/2018.

4 Parker, T. J., Saunders, R. S. & Schneeberger, D. M. Transitional morphology in west Deuteronilus Mensae, Mars: Implications for modification of the lowland/upland boundary. *Icarus* **82**, 111-145 (1989).

5 Parker, T. J., Gorsline, D. S., Saunders, R. S., Pieri, D. C. & Schneeberger, D. M. Coastal geomorphology of the Martian northern plains. *J. Geophys. Res.* **98**, 11061-11078 (1993).

6 Baker, V.R. *et al.* Ancient oceans, ice sheets, and the hydrological cycle on Mars, *Nature* **352**, 589-594 (1991).

7 Head, J. W. *et al.* Possible ancient oceans on Mars: Evidence from Mars Orbiter Laser Altimeter data. *Science* **286**, 2134-2137 (1999).

8 Clifford, S. M. & Parker, T. J. The evolution of the Martian hydrosphere: Implications for the fate of a primordial ocean and the current state of the northern plains. *Icarus* **154**, 40-79 (2001).

9 Kreslavsky, M. A. & Head, J. W. Fate of outflow channel effluent in the northern lowlands of Mars: The Vastitas Borealis Formation as a sublimation residue from frozen ponded bodies of water. *J. Geophys. Res.* **107**, 5121, doi: 10.1029/2001JE001831 (2002).

10 Mouginot, J., Pommerol, A., Beck, P., Kofman, W. & Clifford, S. M. Dielectric map of the Martian northern hemisphere and the nature of plain filling materials. Geophys. Res. Lett. **39**, L02202, doi:[10.1029/2011GL050286](http://dx.doi.org/10.1029/2011GL050286) (2012).

11 Oehler, D.Z. & Allen, C.C. Giant polygons and mounds in the lowlands of Mars: signatures of an ancient ocean? *Astrobiology*, **12(6)**, 601-615 (2012).

12 Moscardelli, L., Dooley, T., Dunlap, D., Jackson, M. & Wood, L. Deep-water polygonal fault systems as terrestrial analogs for large-scale Martian polygonal terrains. *GSA Today* **22**, 4, <http://dx.doi.org/10.1130/GSATG147A.1> (2012).

13 Tanaka, K. L., Skinner, J. A. & Hare, T. M. Geologic map of the northern plains of Mars. U.S. Geological Survey Scientific Investigations Map 2888, scale 1:15,000,000 (1 mm = 15 km) at 90° N and 1:7,500,000 at 0° N, <http://pubs.usgs.gov/sim/2005/2888/> (2005) Date of access: 03/14/2018.

14 Malin, M. C. & Edgett, K. S. Oceans or seas in the Martian northern lowlands: High-resolution imaging tests of proposed coastlines. *Geophys. Res. Lett.* **26**, 3049-3052 (1999).

15 Tanaka, K. L. Sedimentary history and mass flow structures of Chryse and Acidalia Planitiae, Mars. *J. Geophys. Res.***102**, 4131-4149 (1997).

16 Rodriguez, J.A.P. *et al.* Tsunami waves extensively resurfaced the shorelines of an early Martian ocean. *Scientific Reports*, **6**, Article Number 25106, doi:10.1038/srep25106 (2016).

17 Costard, F. *et al.* Modeling tsunami propagation and the emplacement of thumbprint terrain in an early Mars ocean, J*. Geophys. Res. Planets* **122**, 633–649, doi:10.1002/2016JE005230 (2017).

18 Paris, R. *et al.* Tsunamis as geomorphic crises: Lessons from the December 26, 2004 tsunami in Lhok Nga, West Banda Aceh (Sumatra, Indonesia). *Geomorphology* **104**, 59-72 (2009).

19 Goto, K., Hashimoto, K., D., S., Yanagisawa, H. & Abe, T. Spatial thickness variability of the 2011 Tohoku-oki tsunami deposits along the coastline of Sendai Bay. *Mar. Geol.* **358**, 38-48 (2014).

20 Citron, R. I. *et al.* Timing of oceans on Mars from shoreline deformation, *Nature* **555**, 643-646 (2018).

21 Lucchitta, B. K., Isbell, N. K. & Howington-Kraus, A. Topography of Valles Marineris: Implications for erosional and structural history. *J. Geophys. Res.*, **99**, 3783-3798 (1994).

22 Harrison, K.P. & Chapman, M.G. Evidence for ponding and catastrophic floods in central Valles Marineris, Mars. *Icarus*,**198**, 351–364 (2008).

23 Lucchitta, B. K. Lakes in Valles Marineris. In Lakes on Mars, (Eds.) Cabrol, N.A. & E.A. Grin, Elsevier, 111-161, doi: 10.1016/B978-0-444-52854-4.00005-2 (2010).

24 Warner, N. H., Sowe, M., Gupta, S., Dumke, A. & Goddard, K. Fill and spill of giant lakes in the eastern Valles Marineris region of Mars. *Geology*, **41**, 675-678 (2013).

25 Rodriguez, J.A.P., *et al.* Groundwater flow induced collapse and flooding in Noctis Labyrinthus, Mars. *Planet Space Sci.,* **124***,* 1-14, <http://dx.doi.org/10.1016/j.pss.2015.12.009> (2016).

26 Rodriguez, J.A.P., *et al.* Headward growth of chasmata by volatile outbursts, collapse, and drainage, Mars, *Geophys. Res. Lett.*, **33**, L18203, doi:10.1029/2006GL026275 (2006).

27 Nedell, S. S., Squyres, S. W. & Andersen D. W. Origin and evolution of the layered deposits in the Valles Marineris, Mars. *Icarus*, **70**, 3, 409-441, IN1, 415-441 (1987).

28 Malin, M. C. & Edgett, K. S. Sedimentary rocks of early Mars. *Science*, **290**, 5498, 1927-1937 (2000).

29 Malin, M. C. & Edgett, K. S. Mars Global Surveyor Mars Orbiter Camera: Interplanetary cruise through primary mission. *J. Geophys. Res.,* **106**(E10), **23**,429-23,570 (2001).

30 Gendrin, A., *et al.* Sulfates in Martian Layered Terrains: The OMEGA/Mars Express View. *Science*, **307**, 1587-1591 (2005).

31 Murchie, S., *et al.* Evidence for the origin of layered deposits in Candor Chasma, Mars, from mineral composition and hydrologic modeling. *J. Geophys. Res*., **114**, E00D05, doi:10.1029/2009JE003343 (2009).

32 Weitz, C. M., Noe Dobrea, E. Z., Lane, M. D. & Knudson, A. Geologic relationships between gray hematite, sulfates, and clays in Capri Chasma. *J. Geophys. Res.* **117**, E11, 10.1029/2012JE004092 (2012).

33 Rossi, A. P., *et al.* Large-scale spring deposits on Mars? *J. Geophys. Res.,* **113**, E08016, doi:10.1029/2007JE003062 (2008).

34 Andrews-Hanna, J. C., Zuber, M. T., Arvidson, R. E. & Wiseman, S. M. Early Mars hydrology: Meridiani playa deposits and the sedimentary record of Arabia Terra. *J. Geophys. Res*., E06002, doi:10.1029/2009JE003485 (2010).

35 Niles, P. B. & Michalski, J. Meridiani sediments on Mars formed through weathering in massive ice deposits. *Nature Geoscience*, **2**, 215–220, doi:10.1038/NGEO438 (2009).

36 Michalski, J. R. & Niles, P. B. Atmospheric origin of martian interior layered deposits: Links to climate change and the global sulfur cycle. *Geology*, **40**(5), 419-422, doi:10.1130/G32971.1 (2012).

37 Chapman, M. G. & Tanaka, K. L. Interior trough deposits on Mars: Subice volcanoes? *J. Geophys. Res.,* **106**, 10,087-10,100 (2001).

38 Harrison, K. P. & Grimm, R. E. Tharsis recharge: A source of groundwater for Martian outflow channels. *Geophys. Res. Lett*., **31**, L14703 (2004).

39 Hanna, J. C. & Phillips, R. J. Hydrological modeling of the Martian crust with application to the pressurization of aquifers, *J. Geophys. Res*., **110**, E01004, doi:10.1029/2004JE002330 (2005).

40 Harrison, K. P. & Grimm, R. E. Regionally compartmented groundwater flow on Mars. *J. Geophys. Res*., **114**, E04004, doi:10.1029/2008JE003300 (2009).

41 Montgomery, D. R. *et al.* Continental-scale salt tectonics on Mars and the origin of Valles Marineris and associated outflow channels. *Geol. Soc. Am. Bull*., **121**, 117-133. doi:10.1130/B26307.1 (2009).

**The Aral Sea as an Analog to the Inland Sea Supplement**

In the 1960s the Soviet Union diverted the Aral Sea’s fluvial sources, the Syr Darya and Amu Darya Rivers, to nearby areas to supply water to vast irrigation projects^1^. The resulting reduction in inflowing fluvial discharges, along with the regional effect of global climate warming^2^, resulted in the onset of large-scale marine regression driven by high rates of seawater evaporation (Supplement’s Figure 1, panels a-f). The sea’s current extension is less than ~7,000 km^2^, ten times less than in 1969 when it covered ~70,000 km^2^ (3). See animation at <https://earthobservatory.nasa.gov/Features/WorldOfChange/aral_sea.php>.

Wave-cut terraces are absent from the regression exposed eastern coastal plains, which consist of thick deltaic deposits^2, 3^ with slopes that range between 0.01-0.015^o^ (Supplement’s Figure 1, panel g). These shallow slopes caused the seafront over these plains to retreat ~125 km due to a sea elevation drop of ~26 m between 1960 and 2009, averaging ~2.5 km/yr (~7 m/day). Daily changes in shoreline level could have effectively prevented the development of distinct coastal features. Also, intense deflation processes on the flat and dry marine platforms of the eastern Aral Sea^1,3^ and the persistent presence of perennial sea ice during several months a year^4,5^ could have also contributed to the lack of preserved shoreline features.

In contrast to the eastern coastal plains, the sea’s western and northern seafronts exhibit surface slopes that range from 0.5 ~ to ~10^o^ (Supplement’s Figure 1, panel g). In these regions the 26 m sea level drop between 1996 and 2009 caused less than ~1.5 km of shoreline retreat, averaging 30 m/yr. These coastal areas exhibit well-developed sequences of stair-step terraces (e.g., Supplement’s Figure 1, panel h(1)) ascribable to the regression-transgression history during the last 10 kyr^3^. We propose that these terraces and those flanking the margins of some of the Hydraotes Chaos mesas (Supplement’s Figure 1, panel h(2); Fig. 2F; Fig. S4C) formed along steeply inclined submerged scarps due to low regression rates. On the other hand, the rapid regression over shallowly submerged low-inclination marine plains explains the absence of marine terraces around the perimeters of the Aral sea (Supplement’s Figure 1, panel h(3)) and the inland sea (Supplement’s Figure 1, panel h(4); Fig. S4; supplementary materials on the inland sea’s thermal stability).


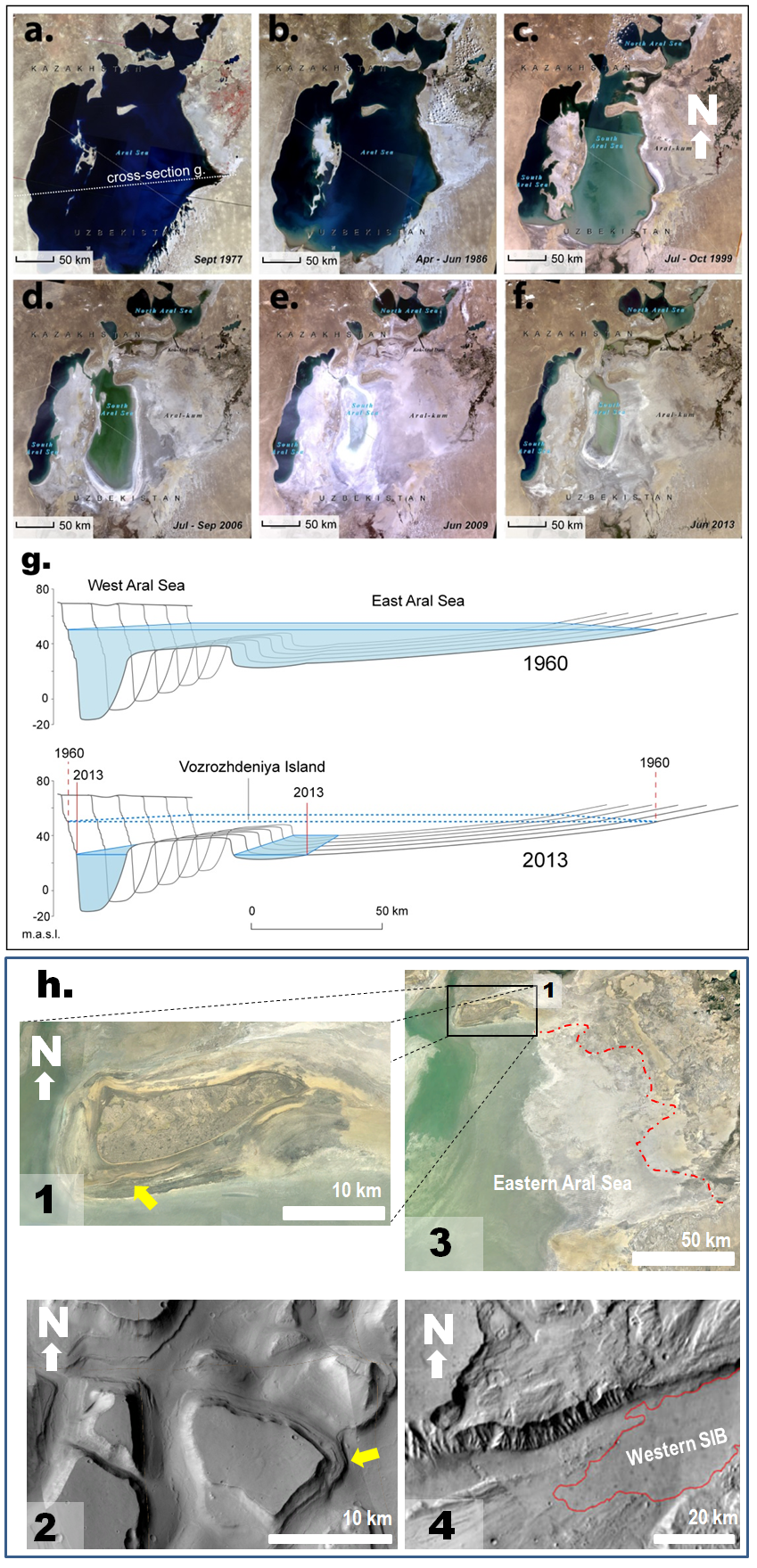


**Supplement’s Figure 1** Aral Sea regression during the last 50 years. **(a to f)** Time-lapse Landsat satellite imagery illustrating the Aral Sea´s retreat from 1977 to 2013. Source: USGS/NASA; visualization by UNEP/GRIDSioux Falls (in <https://na.unep.net/geas/getUNEPPageWithArticleIDScript.php?article_id=108>). **(g)** Historical reconstruction of the sea’s retreat from 1960 to 2013, in response to a 26 m drop in water level, caused by the Soviet irrigation projects and change in global climate. The sea´s bathymetry is modified from Boomer *et al*.^3^ and Singh *et al*.^6^. **(h) (1)** View of the NE Aral Sea showing a steep flanking margin marked by terraces (yellow arrow) (Google Earth view centered at 45°38'47"N, 59°52'41"E). Context box in subpanel 3. **(2)** Similar terraces marking the flanks of a mesa in Hydraotes Chaos (yellow arrow, part of a CTX mosaic centered at 0°43'409"N, 33°24'49"W). **(3)** The red line traces the former extent of the eastern Aral Sea. Instead of terraces, the upper reaches of a sedimentary blanket trace the high sea level (Google Earth view centered at 45° 8'39N, 60°45'41"E). Map data: Google, Image Landsat / Copernicus. Image © 2018 CNES /Airbus. Image © 2018. DigitalGlobe. **(4)** Part of the sedimentary deposit within western SIB (red line, part of a THEMIS daytime IR mosaic centered at 13°13'3"S, 41°10'48"W).

**References cited in the Aral Sea supplement**

1 Micklin, P.P. Desiccation of the Aral Sea: A Water Management Disaster in the Soviet Union. *Science,* **241**, 1170-1176, doi:10.1126/science.241.4870.1170 (1988).

2 Cretaux, J.F., Letolle, R. & Bergé-Nguyen, M. History of Aral Sea level variability and current scientific debates. *Global and Planetary Change,* **110**, 99–113, doi:/10.1016/j.gloplacha.2013.05.006 (2013).

3 Boomer, I., Aladin, N., Plotnikov, I. & Whatley, R. The palaeolimnology of the Aral Sea: a review. *Quaternary Science Reviews,* **19**, 1259-1278 (2000).

4 Kouraev, A.V., *et al.* Sea ice cover in the Caspian and Aral Seas from historical and satellite data. *Journal of Marine Systems,* **47**, 89– 100, doi:10.1016/j.jmarsys.2003.12.011 (2004).

5 Kouraev, A.V., Kostianoy, A.G. & Lebedev, S.A. Recent changes of sea level and ice cover in the Aral Sea derived from satellite data (1992–2006). *Journal of Marine Systems,* **76** (3), 272–286, doi:10.1016/j.jmarsys.2008.03.016 (2009).

6 Singh, A., Kumar, U. & Seitz, F. Remote Sensing of Storage Fluctuations of Poorly Gauged Reservoirs and State Space Model (SSM)-Based Estimation. *Remote Sens.,* **7**, 17113–17134, doi:10.3390/rs71215872 (2015).

**The Inland Sea’s Thermal Stability Supplement**

To perform our inland sea thermal numerical model we used the following initial and boundary conditions:

-Ambient average temperature = 215 K, consistent for near-equatorial average temperature as indicated in Clifford & Parker^1^.

-Initial water temperature (greater than freezing, less than boiling temperature) = 293 K.

-Relative humidity ranging from 20 % (daytime) to 60 % (night-time). Daily variations in relative humidity are large and seasonal variations are significant, mainly reflecting changing ambient temperatures^2^. Various ranges in humidity were considered, e.g., 10% to 90%. Results do not vary greatly since the average humidity over a day does not change much.

-Heat flux from the interior of Mars ~ 30 mW/m^2^. Heat flow estimates cover a range of values, higher in the past, and likely varying over different regions of the planet. Our value is consistent with those presented in Clifford & Parker^1^.

The simulation calculated the thermal diffusion through the depth of the sea at each longitude and latitude pair. It modeled evaporation when the sea surface is liquid and sublimation when the sea surface is frozen, with subsequent lowering of the sea surface. It tracked the freezing interface, taking into account daily and seasonal variations in solar insolation, atmospheric temperature, relative humidity, temperature dependence of ice and water properties, albedo and a nominal wind speed of 3 m/s, as well as black-body radiation from the surface to the atmosphere. Results are reported in Earth years. A successful simulation of the Aral Sea evaporation provided validation of the model.

The simulation tracked the evolution of the inland sea over 1000 years (Supplement’s Figure 1). We found that the combined roles of evaporation and sublimation resulted in a shoreline retreat rate of ~0.5 m/year. In 250 years ~50% of the sea’s surface area and an equivalent depth of ~200 m would have disappeared. Then, in ~1000 years, ~90% of the sea, and an equivalent depth of ~500 m would have disappeared. After ~1000 years the only part of the inland sea remaining would have been Hydraotes Chaos.


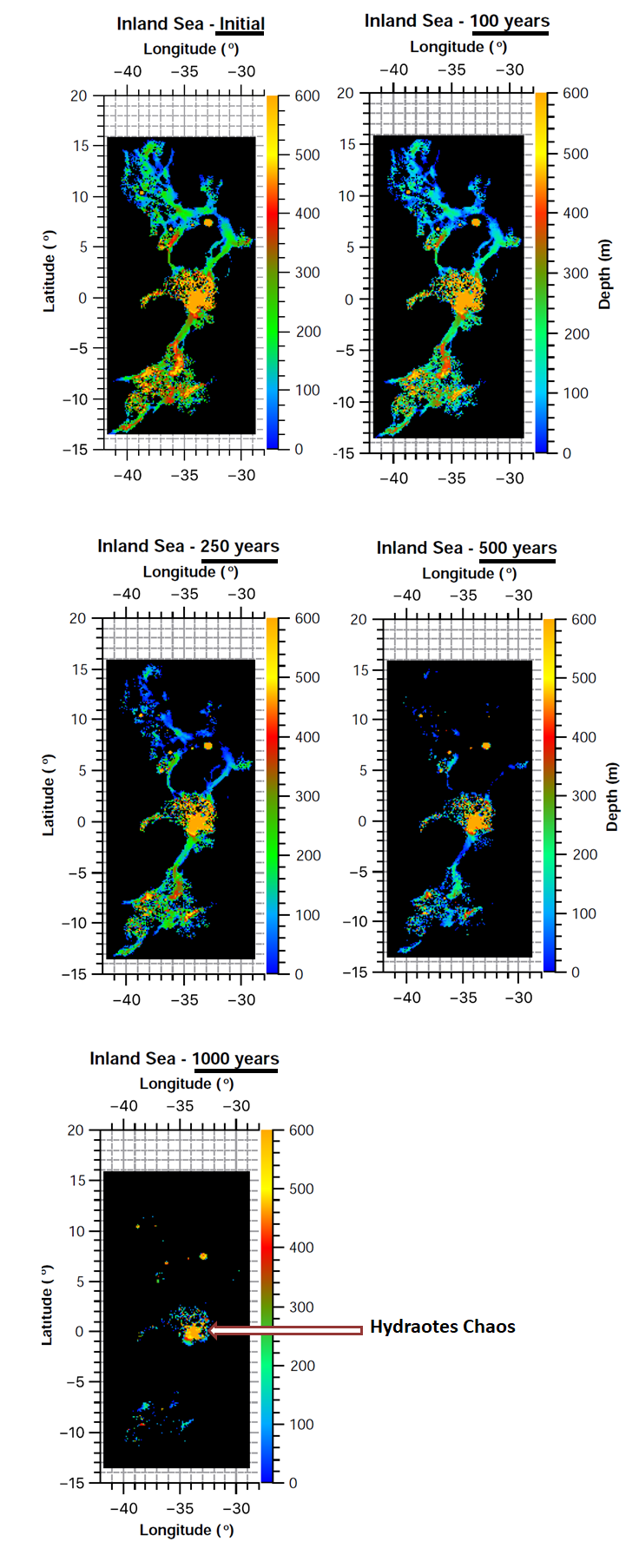
**Supplement’s Fig. 1** The panels show the depth and areal extents of the inland sea at 100, 250, 500, and 1000 years after its emplacement.

The elevation of the frozen-liquid interface at the base of the ice cover would have dropped rapidly initially. However, it would have eventually ended up decreasing at the same rate as the surface sublimation curve (Supplement’s Figure 2), resulting in a constant thickness ice cover (~17 m) over progressively smaller volumes of seawater.

**Supplement’s Fig. 2 (A)** Plot showing the decrease in sea area versus time. **(B)** and **(C)** are plots showing the drop in surface elevation of the sea versus time.  Also shown on the graphs is the depth to the frozen-liquid interface at the base of the ice cover.  By about 100 years after emplacement, the sea’s frozen surface elevation drops at an almost constant rate. Time in Earth years.


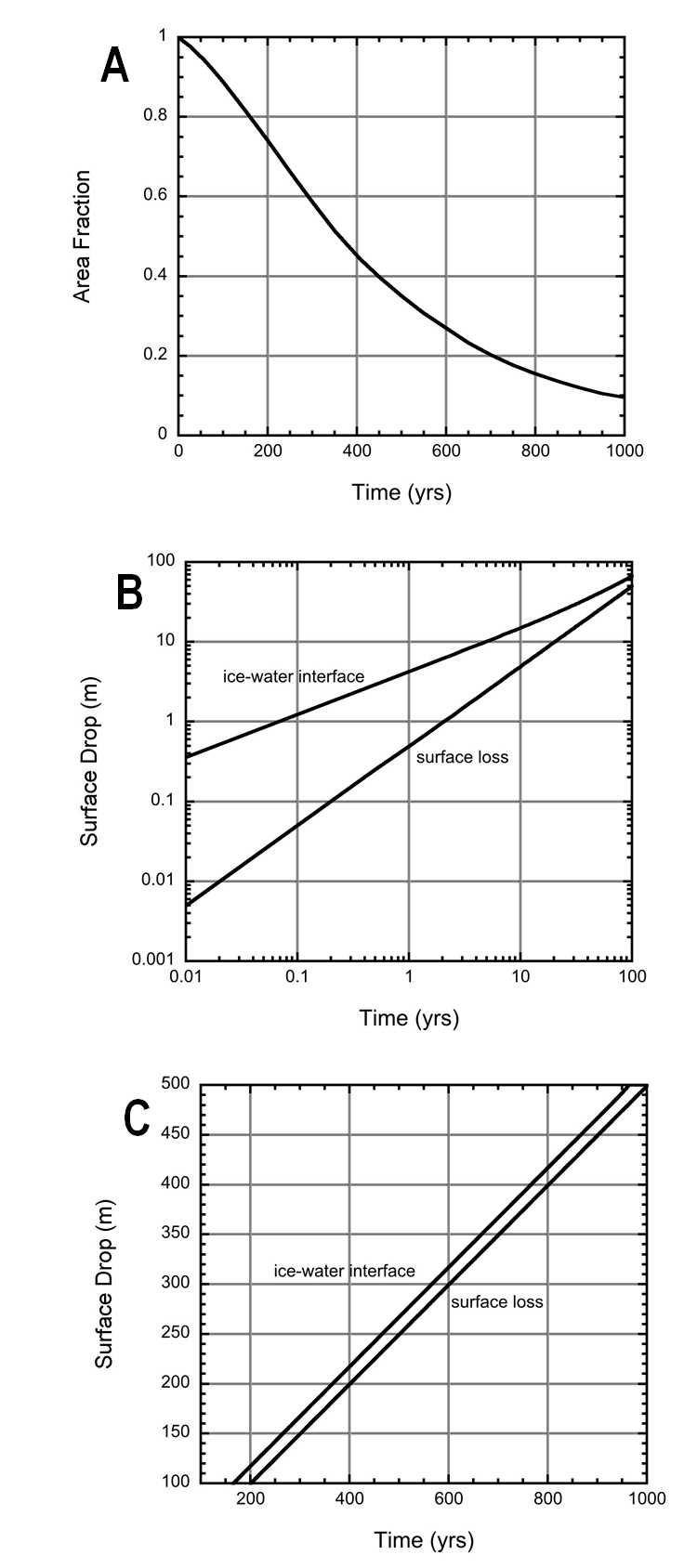

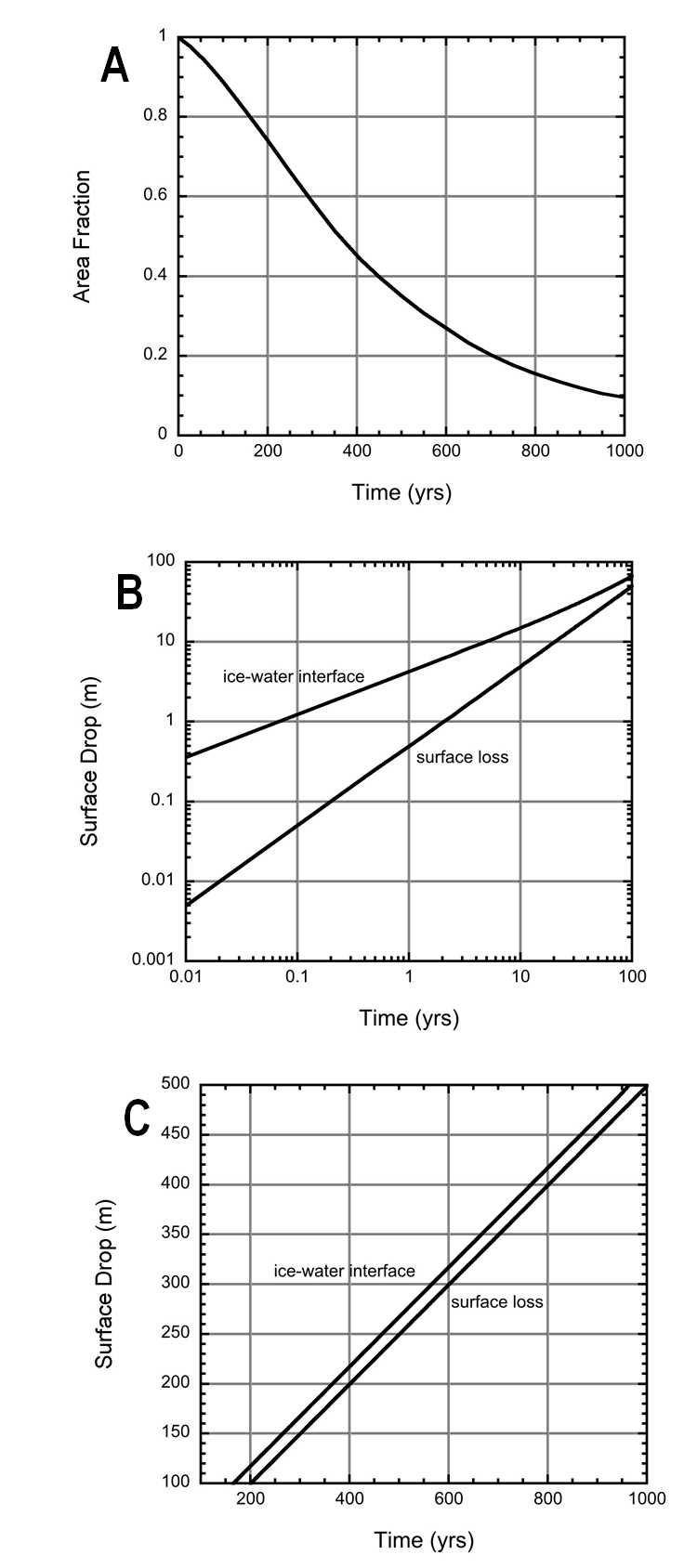

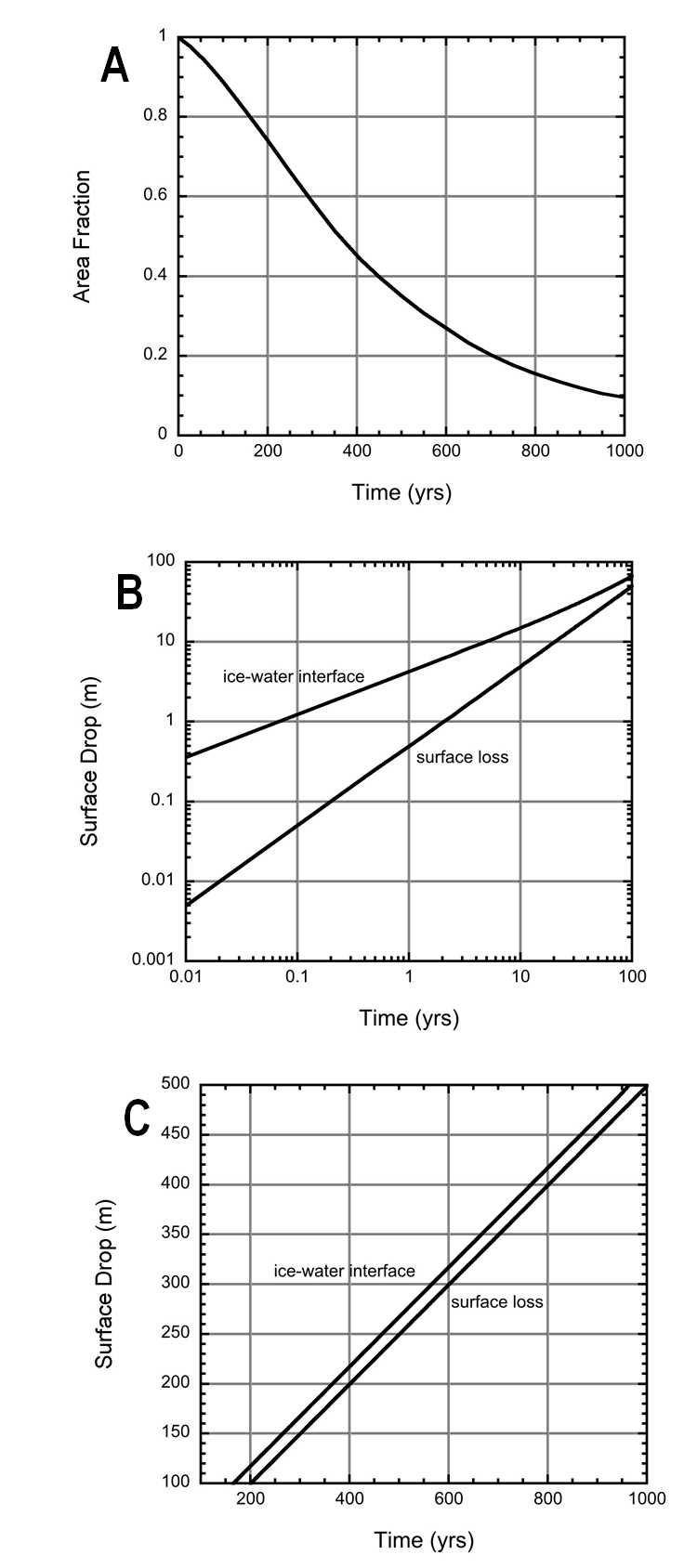


While not modeled in this investigation, another loss mechanism for the inland sea would have been seepage into the underlying regolith, especially if the regolith at the time of sea emplacement was dry and chemically pristine (i.e., no or little clays, which could have reduced permeability). Estimates of permeability and porosity for the upper several hundred meters of Martian regolith^1^ suggest permeabilities ranging from several to 10’s of darcies, and a porosity on the order of 35% at the surface. Porosity would have decayed exponentially with depth, still being on the order of 10% at 1 km depth. Permeability, based on Earth observations, should also decay exponentially^1,3^. Under these conditions, and with the pressure drive from an overlying sea, some of the water could drain into the regolith. Based on simulations with a multi-phase porous flow and transport model^4,5,6^, infiltration into a dry regolith, made up of rocks with temperatures above the water freezing point, could penetrate up to a kilometer of depth in several years. Infiltration into a dry but cryogenic regolith would proceed much more slowly, because of the formation of freezing fronts. Pore clogging by ice formation would greatly decrease the already decreasing (with depth) permeability. Penetration to a few 10s of meters could be possible, though, over many years. Penetration into a cold, ice-filled regolith would be negligible.

All of the above does not consider salts in the sea water. Salts can significantly depress the freezing temperature of a salt solution, (e.g., eutectic temperature is -22^o^ C for NaCl; -52^o^ C for CaCl_2_)^7,8^, and even lower for some ferric chloride/perchlorate salts. Near any freezing front, the liquid solution would concentrate to a eutectic mixture. A salty sea would at least result in a thinner ice cover, than for the pure water case, and even possibly allow an ice-free sea for sufficiently warm (i.e. above eutectic temperature) atmospheric conditions.

**References cited in the thermal stability supplement**

1 Clifford, S. M. & Parker, T. J. The evolution of the martian hydrosphere: Implications for the fate of a primordial ocean and the current state of the northern plains. *Icarus,* **154**, 40-79 (2001).

2 Harri, A. M., *et al*. Mars Science Laboratory relative humidity observations: Initial results, *J. Geophys. Res.* **119**(9), 2132-2147. doi: 10.1002/2013JE004514 (2014).

3 Clifford, S. M. A model for the hydrologic and climatic behavior of water on Mars. *Jour. of Geophys. Res.,* **98**, 10973-11016 (1993).

4 Lewis, K.C., *et al*. Drainage subsidence associated with Arctic permafrost degradation, *Jour. of Geophys. Res. Earth Surface,* **117**(F04019). doi: 10.1029/2011JF002284 (2012).

5 Rowland, J., B.J. Travis, & Wilson, C.J. The role of advective heat transport in talik development beneath lakes and ponds in discontinuous permafrost, *Geophys. Res. Lett*., **38**( L17504). doi:10.1029/2011GL048497 (2011).

6 Travis, B.J. and Rosenberg, N.D. Modeling In Situ Bioremediation of TCE at Savannah River: Effects of Product Toxicity and Microbial Interactions on TCE Degradation, *Environ. Sci. & Tech*., **31**(11), 3093-3102 (1997).

7 Shepherd, T., A. H. Rankin, & Alderton, D. H. M. A Practical Guide to Fluid Inclusion Studies. Blackie & Son, London (1985).

8 Knauth, L. P. & Burt, D. M. Eutectic Brines on Mars: Origin and Possible Relation to Young Seepage Features*, Icarus,* **158**, 267–271. doi:10.1006/icar.2002.6866 (2002).
